# THE CONCISE GUIDE TO PHARMACOLOGY 2017/18: Catalytic receptors

**DOI:** 10.1111/bph.13876

**Published:** 2017-10-21

**Authors:** Stephen PH Alexander, Doriano Fabbro, Eamonn Kelly, Neil V Marrion, John A Peters, Elena Faccenda, Simon D Harding, Adam J Pawson, Joanna L Sharman, Christopher Southan, Jamie A Davies, CGTP Collaborators

**Affiliations:** ^1^ School of Life Sciences University of Nottingham Medical School Nottingham NG7 2UH UK; ^2^ PIQUR Therapeutics Basel 4057 Switzerland; ^3^ School of Physiology, Pharmacology and Neuroscience University of Bristol Bristol BS8 1TD UK; ^4^ Neuroscience Division Medical Education Institute, Ninewells Hospital and Medical School, University of Dundee Dundee DD1 9SY UK; ^5^ Centre for Integrative Physiology University of Edinburgh Edinburgh EH8 9XD UK

## Abstract

The Concise Guide to PHARMACOLOGY 2017/18 provides concise overviews of the key properties of nearly 1800 human drug targets with an emphasis on selective pharmacology (where available), plus links to an open access knowledgebase of drug targets and their ligands (www.guidetopharmacology.org), which provides more detailed views of target and ligand properties. Although the Concise Guide represents approximately 400 pages, the material presented is substantially reduced compared to information and links presented on the website. It provides a permanent, citable, point‐in‐time record that will survive database updates. The full contents of this section can be found at http://onlinelibrary.wiley.com/doi/10.1111/bph.13876/full. Catalytic receptors are one of the eight major pharmacological targets into which the Guide is divided, with the others being: G protein‐coupled receptors, ligand‐gated ion channels, voltage‐gated ion channels, other ion channels, nuclear hormone receptors, enzymes and transporters. These are presented with nomenclature guidance and summary information on the best available pharmacological tools, alongside key references and suggestions for further reading. The landscape format of the Concise Guide is designed to facilitate comparison of related targets from material contemporary to mid‐2017, and supersedes data presented in the 2015/16 and 2013/14 Concise Guides and previous Guides to Receptors and Channels. It is produced in close conjunction with the Nomenclature Committee of the Union of Basic and Clinical Pharmacology (NC‐IUPHAR), therefore, providing official IUPHAR classification and nomenclature for human drug targets, where appropriate.

## Conflict of interest

The authors state that there are no conflicts of interest to declare.

## Overview

Catalytic receptors are cell‐surface proteins, usually dimeric in nature, which encompass ligand binding and functional domains in one polypeptide chain. The ligand binding domain is placed on the extracellular surface of the plasma membrane and separated from the functional domain by a single transmembrane‐spanning domain of 20‐25 hydrophobic amino acids. The functional domain on the intracellular face of the plasma membrane has catalytic activity, or interacts with particular enzymes, giving the superfamily of receptors its name. Endogenous agonists of the catalytic receptor superfamily are peptides or proteins, the binding of which may induce dimerization of the receptor, which is the functional version of the receptor.

Amongst the catalytic receptors, particular subfamilies may be readily identified dependent on the function of the enzymatic portion of the receptor. The smallest group is the particulate guanylyl cyclases of the natriuretic peptide receptor family. The most widely recognized group is probably the receptor tyrosine kinase (RTK) family, epitomized by the neurotrophin receptor family, where a crucial initial step is the activation of a signalling cascade by autophosphorylation of the receptor on intracellular tyrosine residue(s) catalyzed by enzyme activity intrinsic to the receptor. A third group is the extrinsic protein tyrosine kinase receptors, where the catalytic activity resides in a separate protein from the binding site. Examples of this group include the GDNF and ErbB receptor families, where one, catalytically silent, member of the heterodimer is activated upon binding the ligand, causing the second member of the heterodimer, lacking ligand binding capacity, to initiate signaling through tyrosine phosphorylation. A fourth group, the receptor threonine/serine kinase (RTSK) family, exemplified by TGF‐*β* and BMP receptors, has intrinsic serine/threonine protein kinase activity in the heterodimeric functional unit. A fifth group is the receptor tyrosine phosphatases (RTP), which appear to lack cognate ligands, but may be triggered by events such as cell:cell contact and have identified roles in the skeletal, hematopoietic and immune systems.

A further group of catalytic receptors for the Guide is the integrins, which have roles in cell:cell communication, often associated with signaling in the blood.

## Family structure

S226 Cytokine receptor family

S227 IL‐2 receptor family

S228 IL‐3 receptor family

S229 IL‐6 receptor family

S231 IL‐12 receptor family

S232 Prolactin receptor family

S232 Interferon receptor family

S233 IL‐10 receptor family

S234 Immunoglobulin‐like family of IL‐1 receptors

S235 IL‐17 receptor family

S236 GDNF receptor family

S237 Integrins

S241 Natriuretic peptide receptor family

S242 Pattern recognition receptors

S242 Toll‐like receptor family

S244 NOD‐like receptor family

Ű RIG‐I‐like receptor family

Ű Receptor kinases

Ű TK: Tyrosine kinase

S246 Receptor tyrosine kinases (RTKs)

S246 Type I RTKs: ErbB (epidermal growth factor) receptor family

S247 Type II RTKs: Insulin receptor family

S248 Type III RTKs: PDGFR, CSFR, Kit, FLT3 receptor family

S250 Type IV RTKs: VEGF (vascular endothelial growth factor) receptor family

S250 Type V RTKs: FGF (fibroblast growth factor) receptor family

S251 Type VI RTKs: PTK7/CCK4

S252 Type VII RTKs: Neurotrophin receptor/Trk family

S253 Type VIII RTKs: ROR family

S253 Type IX RTKs: MuSK

S254 Type X RTKs: HGF (hepatocyte growth factor) receptor family

S255 Type XI RTKs: TAM (TYRO3‐, AXL‐ and MER‐TK) receptor family

S255 Type XII RTKs: TIE family of angiopoietin receptors

S256 Type XIII RTKs: Ephrin receptor family

S256 Type XIV RTKs: RET

S257 Type XV RTKs: RYK

S258 Type XVI RTKs: DDR (collagen receptor) family

S258 Type XVII RTKs: ROS receptors

S258 Type XVIII RTKs: LMR family

S259 Type XIX RTKs: Leukocyte tyrosine kinase (LTK) receptor family

S260 Type XX RTKs: STYK1

Ű TKL: Tyrosine kinase‐like

S260 Receptor serine/threonine kinase (RSTK) fam‐ily

S261 Type I receptor serine/threonine kinases

S261 Type II receptor serine/threonine kinases

S262 Type III receptor serine/threonine kinases

S262 RSTK functional heteromers

S264 Receptor tyrosine phosphatase (RTP) family

S265 Tumour necrosis factor (TNF) receptor family

## Cytokine receptor family

### Overview

Cytokines are not a clearly defined group of agents, other than having an impact on immune signalling pathways, although many cytokines have effects on other systems, such as in development. A feature of some cytokines, which allows them to be distinguished from hormones, is that they may be produced by “non‐secretory” cells, for example, endothelial cells. Within the cytokine receptor family, some subfamilies may be identified, which are described elsewhere in the Guide to PHARMACOLOGY, receptors for the TNF family, the TGF‐*β* family and the chemokines. Within this group of records are described Type I cytokine receptors, typified by interleukin receptors, and Type II cytokine receptors, exemplified by interferon receptors. These receptors possess a conserved extracellular region, known as the cytokine receptor homology domain (CHD), along with a range of other structural modules, including extracellular immunoglobulin (Ig)‐like and fibronectin type III (FBNIII)‐like domains, a transmembrane domain, and intracellular homology domains. An unusual feature of this group of agents is the existence of soluble and decoy receptors. These bind cytokines without allowing signalling to occur. A further attribute is the production of endogenous antagonist molecules, which bind to the receptors selectively and prevent signalling. A commonality of these families of receptors is the ligand‐induced homo‐ or hetero‐oligomerisation, which results in the recruitment of intracellular protein partners to evoke cellular responses, particularly in inflammatory or haematopoietic signalling. Although not an exclusive signalling pathway, a common feature of the majority of cytokine receptors is activation of the JAK/STAT pathway. This cascade is based around the protein tyrosine kinase activity of the Janus kinases (JAK), which phosphorylate the receptor and thereby facilitate the recruitment of signal transducers and activators of transcription (STATs). The activated homo‐ or heterodimeric STATs function principally as transcription factors in the nucleus.

**T**ype I cytokine receptors are characterized by two pairs of conserved cysteines linked via disulfide bonds and a C‐terminal WSXWS motif within their CHD. Type I receptors are commonly classified into five groups, based on sequence and structual homology of the receptor and its cytokine ligand, which is potentially more reflective of evolutionary relationships than an earlier scheme based on the use of common signal transducing chains within a receptor complex.

Type II cytokine receptors also have two pairs of conserved cysteines but with a different arrangement to Type I and also lack the WSXWS motif.

## IL‐2 receptor family

### Overview

The IL‐2 receptor family consists of one or more ligand‐selective subunits, and a common *γ* chain (*γ*c): IL2RG, P31785), though IL‐4 and IL‐7 receptors can form complexes with other receptor chains. Receptors of this family associate with Jak1 and Jak3, primarily activating Stat5, although certain family members can also activate Stat1, Stat3, or Stat6. Ro264550 has been described as a selective IL‐2 receptor antagonist, which binds to IL‐2 [204].

**Table:** 

Nomenclature	Interleukin‐2 receptor	Interleukin‐4 receptor type I	Interleukin‐4 receptor type II	Interleukin‐7 receptor	Interleukin‐9 receptor
Subunits	Interleukin‐2 receptor subunit *β* (Ligand‐binding subunit), Interleukin‐2 receptor subunit *γ* (Other subunit), Interleukin‐2 receptor subunit *α* (Ligand‐binding subunit)	Interleukin‐4 receptor subunit *α* (Ligand‐binding subunit), Interleukin‐2 receptor subunit *γ* (Other subunit)	Interleukin‐13 receptor subunit *α*1 (Other subunit), Interleukin‐4 receptor subunit *α* (Ligand‐binding subunit)	Interleukin‐2 receptor subunit *γ* (Other subunit), Interleukin‐7 receptor subunit *α* (Ligand‐binding subunit)	Interleukin‐2 receptor subunit *γ* (Other subunit), Interleukin 9 receptor (Ligand‐binding subunit)
Endogenous agonists	IL‐2 (IL2, P60568)	IL‐4 (IL4, P05112)	IL‐13 (IL13, P35225), IL‐4 (IL4, P05112)	IL‐7 (IL7, P13232)	IL‐9 (IL9, P15248)
Endogenous antagonists	IL‐1 receptor antagonist (IL1RN, P18510)	‐	‐	‐	‐
Selective antagonists	AF12198 [1]	‐	‐	‐	‐

**Table:** 

Nomenclature	Interleukin 13 receptor, *α*2	Interleukin‐15 receptor	Interleukin‐21 receptor	Thymic stromal lymphopoietin receptor
HGNC, UniProt	IL13RA2, Q14627	–	–	–
Subunits	–	Interleukin‐2 receptor subunit *β* (Ligand‐binding subunit), Interleukin‐15 receptor subunit *α* (Ligand‐binding subunit), Interleukin‐2 receptor subunit *γ* (Other subunit)	Interleukin‐2 receptor subunit *γ* (Other subunit), Interleukin 21 receptor (Ligand‐binding subunit)	Cytokine receptor‐like factor 2 (Other subunit), Interleukin‐7 receptor subunit *α* (Ligand‐binding subunit)
Endogenous agonists	–	IL‐15 (IL15, P40933)	IL‐21 (IL21, Q9HBE4)	thymic stromal lymphopoietin (TSLP, Q969D9)
Comments	Decoy receptor that binds IL‐13 (IL13, P35225) as a monomer.	–	–	–

**Table:** 

Nomenclature	Interleukin‐2 receptor subunit *α*	Interleukin‐2 receptor subunit *β*	Interleukin‐2 receptor subunit *γ*	Interleukin‐4 receptor subunit *α*	Interleukin‐7 receptor subunit *α*
HGNC, UniProt	IL2RA, P01589	IL2RB, P14784	IL2RG, P31785	IL4R, P24394	IL7R, P16871
Antibodies	daclizumab (p*K*_d_>8) [176], basiliximab	–	–	dupilumab (pIC_50_ 11.1) [140]	–

**Table:** 

Nomenclature	Interleukin 9 receptor	Interleukin‐13 receptor subunit *α*1	Interleukin‐15 receptor subunit *α*	Interleukin 21 receptor	Cytokine receptor‐like factor 2
HGNC, UniProt	IL9R, Q01113	IL13RA1, P78552	IL15RA, Q13261	IL21R, Q9HBE5	CRLF2, Q9HC73

## IL‐3 receptor family

### Overview

The IL‐3 receptor family signal through a receptor complex comprising of a ligand‐specific *α* subunit and a common *β* chain (CSF2RB, P32927), which is associated with Jak2 and signals primarily through Stat5.

**Table:** 

Nomenclature	Interleukin‐3 receptor	Interleukin‐5 receptor	Granulocyte macrophage colony‐stimulating factor receptor
Subunits	Interleukin 3 receptor, *α* subunit (Ligand‐binding subunit), Cytokine receptor common *β* subunit (Other subunit)	Interleukin 5 receptor, *α* subunit (Ligand‐binding subunit), Cytokine receptor common *β* subunit (Other subunit)	GM‐CSF receptor, *α* subunit (Ligand‐binding subunit), Cytokine receptor common *β* subunit (Other subunit)
Endogenous agonists	IL‐3 (IL3, P08700)	IL‐5 (IL5, P05113)	G‐CSF (CSF3, P09919), GM‐CSF (CSF2, P04141)
Selective antagonists	–	YM90709 [134]	–

### Subunits

**Table:** 

Nomenclature	Interleukin 3 receptor, *α* subunit	Interleukin 5 receptor, *α* subunit	GM‐CSF receptor, *α* subunit	Cytokine receptor common *β* subunit
HGNC, UniProt	IL3RA, P26951	IL5RA, Q01344	CSF2RA, P15509	CSF2RB, P32927
Endogenous agonists	IL‐3 (IL3, P08700)	IL‐5 (IL5, P05113)	GM‐CSF (CSF2, P04141)	–
Antibodies	–	benralizumab (p*K*_d_ 8.7) [109]	mavrilimumab (pIC_50_ 9.9) [32]	–

## IL‐6 receptor family

### Overview

The IL‐6 receptor family signal through a ternary receptor complex consisting of the cognate receptor and either the IL‐6 signal transducer gp130 (IL6ST, P40189) or the oncostatin M‐specific receptor, *β* subunit (OSMR, Q99650), which then activates the JAK/STAT, Ras/Raf/MAPK and PI 3‐kinase/PKB signalling modules. Unusually amongst the cytokine receptors, the CNTF receptor is a glycerophosphatidylinositol‐linked protein.

**Table:** 

Nomenclature	Interleukin‐6 receptor	Interleukin‐11 receptor	Interleukin‐27 receptor	Interleukin‐31 receptor	Ciliary neutrophic factor receptor	Leukemia inhibitory factor receptor	Oncostatin‐M receptor
Subunits	Interleukin‐6 receptor, *α* subunit (Ligand‐binding subunit), Interleukin‐6 receptor, *β* subunit (Other subunit)	Interleukin‐11 receptor, *α* subunit (Ligand‐binding subunit), Interleukin‐6 receptor, *β* subunit (Other subunit)	Interleukin‐6 receptor, *β* subunit (Other subunit), Interleukin 27 receptor, alpha (Ligand‐binding subunit)	Interleukin‐31 receptor, *α* subunit (Ligand‐binding subunit), Oncostatin M‐ specific receptor, *β* subunit (Other subunit)	Leukemia inhibitory factor receptor (Other subunit), Interleukin‐6 receptor, *β* subunit, Ciliary neurotrophic factor receptor *α* subunit (Ligand‐binding subunit)	Leukemia inhibitory factor receptor (Ligand‐binding subunit), Interleukin‐6 receptor, *β* subunit (Other subunit)	Interleukin‐6 receptor, *β* subunit (Other subunit), Oncostatin M‐specific receptor, *β* subunit (Ligand‐binding subunit)
Endogenous agonists	IL‐6 (IL6, P05231) [157]	IL‐11 (IL11, P20809)	IL‐27 (EBI3 IL27, Q14213 Q8NEV9)	IL‐31 (IL31, Q6EBC2)	CRCF1/CLCF1 heterodimer (CLCF1 CRLF1, O75462 Q9UBD9), ciliary neurotrophic factor (CNTF, P26441)	LIF (LIF, P15018), cardiotrophin‐1 (CTF1, Q16619), oncostatin M (OSM, P13725)	oncostatin M (OSM, P13725)
Agonists	–	oprelvekin [10, 195]	–	–	–	–	–
Antibodies	vobarilizumab (p*K*_d_ 12.7) [185], sapelizumab (p*K*_d_ 8.9) [92], tocilizumab (p*K*_d_ 8.6)	–	–	–	–	–	–

### Subunits

**Table:** 

Nomenclature	Interleukin‐6 receptor, *α* subunit	Interleukin‐6 receptor, *β* subunit
Systematic nomenclature	interleukin 6 receptor	interleukin 6 signal transducer
HGNC, UniProt	IL6R, P08887	IL6ST, P40189
Common abreviation	IL6R	IL6ST
Endogenous agonists	IL‐6 (IL6, P05231) [ 157]	–
Antibodies	sarilumab (Binding) (p*K*_d_ 10.6–11.1) [198]	–

**Table:** 

Nomenclature	Interleukin‐11 receptor, *α* subunit	Interleukin 27 receptor, alpha	Interleukin‐31 receptor, *α* subunit	Ciliary neurotrophic factor receptor *α* subunit	Leptin receptor	Leukemia inhibitory factor receptor	Oncostatin M‐specific receptor, *β* subunit
HGNC, UniProt	IL11RA, Q14626	IL27RA, Q6UWB1	IL31RA, Q8NI17	CNTFR, P26992	LEPR, P48357	LIFR, P42702	OSMR, Q99650
Endogenous agonists	–	–	–	–	leptin (LEP, P41159) [187] – Mouse	–	–

### Further reading on IL‐6 receptor family

Ho, LJ et al. (2015) Biological effects of interleukin‐6: Clinical applications in autoimmune diseases and cancers. Biochem Pharmacol 97: 16‐26 [PMID:26080005]

Rothaug, M et al. (2016) The role of interleukin‐6 signaling in nervous tissue. Biochim Biophys Acta 1863: 1218‐27 [PMID:27016501]

## IL‐12 receptor family

### Overview

IL‐12 receptors are a subfamily of the IL‐6 receptor family. IL12RB1 is shared between receptors for IL‐12 and IL‐23; the functional agonist at IL‐12 receptors is a heterodimer of IL‐12A/IL‐12B, while that for IL‐23 receptors is a heterodimer of IL‐12B/IL‐23A.

**Table:** 

Nomenclature	Interleukin‐12 receptor	Interleukin‐23 receptor	Interleukin‐12 receptor, *β*1 subunit	Interleukin‐12 receptor, *β*2 subunit	Interleukin 23 receptor
HGNC, UniProt	–	–	IL12RB1, P42701	IL12RB2, Q99665	IL23R, Q5VWK5
Subunits	Interleukin‐12 receptor, *β*2 subunit (Other subunit), Interleukin‐12 receptor, *β*1 subunit (Ligand‐binding subunit)	Interleukin 23 receptor (Ligand‐binding subunit), Interleukin‐12 receptor, *β*1 subunit (Ligand‐binding subunit)	–	–	–
Endogenous agonists	IL‐12 (IL12A IL12B, P29459 P29460)	IL‐23 (IL12B IL23A, P29460)	–	–	–

## Prolactin receptor family

### Overview

Prolactin family receptors form homodimers in the presence of their respective ligands, associate exclusively with Jak2 and signal via Stat5.

**Table:** 

Nomenclature	Eythropoietin receptor	Granulocyte colony‐stimulating factor receptor	Growth hormone receptor	Prolactin receptor	Thrombopoietin receptor
HGNC, UniProt	EPOR, P19235	CSF3R, Q99062	GHR, P10912	PRLR, P16471	MPL, P40238
Endogenous agonists	erythropoietin (EPO, P01588) [44]	G‐CSF (CSF3, P09919)	growth hormone 1 (GH1, P01241), growth hormone 2 (GH2, P01242)	prolactin (PRL, P01236) [46] – Mouse, choriomammotropin (CSH1 CSH2, P01243), chorionic somatomammotropin hormone‐like 1 (CSHL1, Q14406)	thrombopoietin (THPO, P40225)
Agonists	peginesatide [44]	pegfilgrastim	–	–	romiplostim
Selective agonists	–	–	–	–	eltrombopag [119]
Antagonists	–	–	pegvisomant [179]	–	–

### Further reading on Prolactin receptor family

Cabrera‐Reyes, EA et al. (2017) Prolactin function and putative expression in the brain. Endocrine [PMID:28634745]

Goffin, V. (2017) Prolactin receptor targeting in breast and prostate cancers: New insights into an old challenge. Pharmacol Ther [PMID:28549597]

## Interferon receptor family

### Overview

The interferon receptor family includes receptors for type I (*α*, *β* *κ* and *ω*) and type II (*γ*) interferons. There are at least 13 different genes encoding IFN‐*α* subunits in a cluster on human chromosome 9p22: *α*1 (IFNA1, P01562), *α*2 (IFNA2, P01563), *α*4 (IFNA4, P05014), *α*5 (IFNA5, P01569), *α*6 (IFNA6, P05013), *α*7 (IFNA7, P01567), *α*8 (IFNA8, P32881), *α*10 (IFNA10, P01566), *α*13 (IFNA13, P01562), *α*14 (IFNA14, P01570), *α*16 (IFNA16, P05015), *α*17 (IFNA17, P01571) and *α*21 (IFNA21, P01568).

**Table:** 

Nomenclature	Interferon‐*α*/*β* receptor	Interferon‐*γ* receptor
Subunits	Interferon *α*/*β* receptor 2 (Other subunit), interferon *α*/*β* receptor 1 (Ligand‐binding subunit)	Interferon *γ* receptor 2 (Other subunit), Interferon *γ* receptor 1 (Ligand‐binding subunit)
Endogenous agonists	IFN‐*α*1/13 (IFNA1 IFNA13, P01562), IFN‐*α*10 (IFNA10, P01566), IFN‐*α*14 (IFNA14, P01570), IFN‐*α*16 (IFNA16, P05015), IFN‐*α*17 (IFNA17, P01571), IFN‐*α*2 (IFNA2, P01563), IFN‐*α*21 (IFNA21, P01568), IFN‐*α*4 (IFNA4, P05014), IFN‐*α*5 (IFNA5, P001569), IFN‐*α*6 (IFNA6, P05013), IFN‐*α*7 (IFNA7, P01567), IFN‐*α*8 (IFNA8, P32881), IFN‐*β* (IFNB1, P01574), IFN‐*κ* (IFNK, Q9P0W0), IFN‐*ω* (IFNW1, P05000)	IFN‐*γ* (IFNG, P01579)
Selective agonists	peginterferon alfa‐2b [191]	–

### Subunits

**Table:** 

Nomenclature	interferon *α*/*β* receptor 1	Interferon *α*/*β* receptor 2	Interferon *γ* receptor 1	Interferon *γ* receptor 2
HGNC, UniProt	IFNAR1, P17181	IFNAR2, P48551	IFNGR1, P15260	IFNGR2, P38484
Selective agonists	peginterferon alfa‐2b [191]	–	–	–
Antibodies	anifrolumab (p*K*_d_>10) [24]	–	–	–

### Further reading on Interferon receptor family

Kotenko, SV et al. (2017) Contribution of type III interferons to antiviral immunity: location, location, location. J Biol Chem 292: 7295‐7303 [PMID:28289095]

Ng, CT, et al. (2016) Alpha and Beta Type 1 Interferon Signaling: Passage for Diverse Biologic Outcomes. Cell 164: 349‐52 [PMID:26824652]

Schreiber, G. (2017) The molecular basis for differential type I interferon signaling. J Biol Chem 292: 7285‐7294 [PMID:28289098]

## IL‐10 receptor family

### Overview

The IL‐10 family of receptors are heterodimeric combinations of family members: IL10RA/IL10RB responds to IL‐10; IL20RA/IL20RB responds to IL‐19, IL‐20 and IL‐24; IL22RA1/IL20RB responds to IL‐20 and IL‐24; IL22RA1/IL10RB responds to IL‐22; IFNLR1(previouly known as IL28RA)/IL10RB responds to IFN‐*λ*1, ‐*λ*2 and ‐*λ*3 (previouly known as IL‐29, IL‐28A and IL‐28B respectively).

**Table:** 

Nomenclature	Interleukin‐10 receptor	Interleukin‐20 receptor	Interleukin‐22*α*1/20*β* heteromer	Interleukin‐22*α*1/10*β* heteromer	Interleukin‐22 receptor *α*2	Interferon‐*λ* receptor 1
HGNC, UniProt	–	–	–	–	IL22RA2, Q969J5	–
Subunits	Interleukin 10 receptor, *α* subunit (Ligand‐binding subunit), Interleukin 10 receptor, *β* subunit (Other subunit)	Interleukin 20 receptor, *β* subunit (Other subunit), Interleukin 20 receptor, *α* subunit (Ligand‐binding subunit)	Interleukin 22 receptor, *α*1 subunit (Ligand‐binding subunit), Interleukin 20 receptor, *β* subunit (Ligand‐binding subunit)	Interleukin 22 receptor, *α*1 subunit (Ligand‐binding subunit), Interleukin 10 receptor, *β* subunit (Ligand‐binding subunit)	–	Interferon‐*λ* receptor subunit 1 (Ligand‐binding subunit), Interleukin 10 receptor, *β* subunit (Other subunit)
Endogenous agonists	IL‐10 (IL10, P22301)	IL‐19 (IL19, Q9UHD0), IL‐20 (IL20, Q9NYY1), IL‐24 (IL24, Q13007)	IL‐20 (IL20, Q9NYY1), IL‐24 (IL24, Q13007)	IL‐22 (IL22, Q9GZX6)	–	IFN‐*λ*1 (IFNL1, Q8IU54), IFN‐*λ*2 (IFNL2, Q8IZJ0), IFN‐*λ*3 (IFNL3, Q8IZI9)
Comments	–	–	–	–	Soluble decoy receptor that binds IL‐22 (IL22, Q9GZX6) as a monomer.	–

### Subunits

**Table:** 

Nomenclature	Interleukin 10 receptor, *α* subunit	Interleukin 10 receptor, *β* subunit	Interleukin 20 receptor, *α* subunit	Interleukin 20 receptor, *β* subunit	Interleukin 22 receptor, *α*1 subunit	Interferon‐*λ* receptor subunit 1
HGNC, UniProt	IL10RA, Q13651	IL10RB, Q08334	IL20RA, Q9UHF4	IL20RB, Q6UXL0	IL22RA1, Q8N6P7	IFNLR1, Q8IU57

### Further reading on IL‐10 receptor family

Felix J et al. (2017) Mechanisms of immunomodulation by mammalian and viral decoy receptors: insights from structures. Nat. Rev. Immunol. 17: 112‐129 [PMID:28028310]

## Immunoglobulin‐like family of IL‐1 receptors

### Overview

The immunoglobulin‐like family of IL‐1 receptors are heterodimeric receptors made up of a cognate receptor subunit and an IL‐1 receptor accessory protein, IL1RAP(Q9NPH3, also known as C3orf13, IL‐1RAcP, IL1R3). They are characterised by extracellular immunoglobulin‐like domains and an intracellular Toll/Interleukin‐1R (TIR) domain.

**Table:** 

Nomenclature	Interleukin‐1 receptor, type I	Interleukin‐33 receptor	Interleukin‐36 receptor	Interleukin‐1 receptor, type II	Interleukin‐18 receptor
Subunits	IL‐1 receptor accessory protein (Other subunit), Interleukin 1 receptor, type I (Ligand‐binding subunit)	IL‐1 receptor accessory protein (Other subunit), Interleukin‐1 receptor‐like 1 (Ligand‐binding subunit)	IL‐1 receptor accessory protein (Other subunit), Interleukin‐1 receptor‐like 2 (Ligand‐binding subunit)	IL‐1 receptor accessory protein (Other subunit), Interleukin 1 receptor, type II (Ligand‐binding subunit)	IL‐18 receptor accessory protein (Other subunit), Interleukin‐18 1 (Ligand‐binding subunit)
Inhibitors	anakinra (p*K*_d_ 7.8) [39]	–	–	–	–
Endogenous agonists	IL‐1*α* (IL1A, P01583), IL‐1*β* (IL1B, P01584)	IL‐33 (IL33, O95760)	IL‐36*α* (IL36A, Q9UHA7), IL‐36*β* (IL36B, Q9NZH7), IL‐36*γ* (IL36G, Q9NZH8)	–	IL‐18 (IL18, Q14116), IL‐37 (IL37, Q9NZH6)
Endogenous antagonists	IL‐1 receptor antagonist (IL1RN, P18510)	–	IL‐36 receptor antagonist (IL36RN, Q9UBH0)	–	–
Selective antagonists	AF12198 [1]	–	–	–	–
Comments	–	–	IL‐36 receptor antagonist (IL36RN, Q9UBH0) is a highly selective antagonist of the response to IL‐36*γ* (IL36G, Q9NZH8).	Decoy receptor that binds IL‐1*α* (IL1A, P01583), IL‐1*β* (IL1B, P01584) and IL‐1 receptor antagonist (IL1RN, P18510).	–

**Table:** 

Nomenclature	Interleukin 1 receptor, type I	Interleukin 1 receptor, type II	Interleukin‐1 receptor‐like 1	Interleukin‐1 receptor‐like 2	Interleukin‐18 1
HGNC, UniProt	IL1R1, P14778	IL1R2, P27930	IL1RL1, Q01638	IL1RL2, Q9HB29	IL18R1, Q13478

## IL‐17 receptor family

### Overview

:

The IL17 cytokine family consists of six ligands (IL‐17A‐F), which signal through five receptors (IL‐17RA‐E).

**Table:** 

Nomenclature	Interleukin‐17 receptor	Interleukin‐25 receptor	Interleukin‐17C receptor
Subunits	Interleukin 17 receptor A (Ligand‐binding subunit), interleukin 17 receptor C (Other subunit)	Interleukin 17 receptor B (Ligand‐binding subunit), Interleukin 17 receptor A (Other subunit)	Interleukin 17 receptor A (Other subunit), Interleukin 17 receptor E (Ligand‐binding subunit)
Endogenous agonists	IL‐17A (IL17A, Q16552), IL‐17A/IL‐17F (IL17A IL17F, Q16552 Q96PD4), IL‐17F (IL17F, Q96PD4)	IL‐17B (IL17B, Q9UHF5), IL‐25 (IL25, Q9H293)	IL‐17C (IL17C, Q9P0M4)

### Subunits

**Table:** 

Nomenclature	Interleukin 17 receptor A	Interleukin 17 receptor B	interleukin 17 receptor C	Interleukin‐17 receptor D	Interleukin 17 receptor E
HGNC, UniProt	IL17RA, Q96F46	IL17RB, Q9NRM6	IL17RC, Q8NAC3	IL17RD, Q8NFM7	IL17RE, Q8NFR9
Antibodies	brodalumab (Binding) (p*K*_d_ 9.2) [206]	–	–	–	–
Comments	–	–	–	The endogenous agonist for this receptor is unknown.	–

### Further reading on IL‐17 receptor family

Beringer, A et al. (2016) IL‐17 in Chronic Inflammation: From Discovery to Targeting. Trends Mol Med 22: 230‐41 [PMID:26837266]

Lubberts, E. (2015) The IL‐23‐IL‐17 axis in inflammatory arthritis. Nat Rev Rheumatol 11: 415‐29 [PMID:25907700]

## GDNF receptor family

### Overview

GDNF family receptors (provisional nomenclature) are extrinsic tyrosine kinase receptors. Ligand binding to the extracellular domain of the glycosylphosphatidylinositol‐linked cell‐surface receptors (tabulated below) activates a transmembrane tyrosine kinase enzyme, RET(see Receptor Tyrosine Kinases). The endogenous ligands are typically dimeric, linked through disulphide bridges: glial cell‐derived neurotrophic factor GDNF(GDNF, P39905) (211 aa); neurturin(NRTN, Q99748) (197 aa); artemin(ARTN, Q5T4W7) (237 aa) and persephin(PSPN, O60542) (PSPN, 156 aa).

**Table:** 

Nomenclature	GDNF family receptor *α*1	GDNF family receptor *α*2	GDNF family receptor *α*3	GDNF family receptor *α*4
HGNC, UniProt	GFRA1, P56159	GFRA2, O00451	GFRA3, O60609	GFRA4, Q9GZZ7
Common abreviation	GFR*α*1	GFR*α*2	GFR*α*3	GFR*α*4
Potency order	GDNF (GDNF, P39905) >neurturin (NRTN, Q99748) >artemin (ARTN, Q5T4W7)	neurturin (NRTN, Q99748) >GDNF (GDNF, P39905)	artemin (ARTN, Q5T4W7)	persephin (PSPN, O60542)
Labelled ligands	[^125^I]GDNF (rat) (p*K*_d_ 10.2–11.5) [90, 180]	–	–	–

### Comments

Inhibitors of other receptor tyrosine kinases, such as semaxanib, which inhibits VEGF receptor function, may also inhibit Ret function [132]. Mutations of RET and GDNF genes may be involved in Hirschsprung's disease, which is characterized by the absence of intramural ganglion cells in the hindgut, often resulting in intestinal obstruction.

### Further reading on GDNF receptor family

Allen SJ et al. (2013) GDNF, NGF and BDNF as therapeutic options for neurodegeneration. Pharmacol. Ther. 138: 155‐75 [PMID:23348013]

Ibanez CF et al. (2017) Biology of GDNF and its receptors ‐ Relevance for disorders of the central nervous system. Neurobiol Dis 97: 80‐89 [PMID:26829643]

Merighi A. (2016) Targeting the glial‐derived neurotrophic factor and related molecules for controlling normal and pathologic pain. Expert Opin Ther Targets 20: 193‐208 [PMID:26863504]

## Integrins

### Overview

Integrins are unusual signalling proteins that function to signal both from the extracellular environment into the cell, but also from the cytoplasm to the external of the cell. The intracellular signalling cascades associated with integrin activation focus on protein kinase activities, such as focal adhesion kinase and Src. Based on this association between extracellular signals and intracellular protein kinase activity, we have chosen to include integrins in the ‘Catalytic receptors’ section of the database until more stringent criteria from NC‐IUPHAR allows precise definition of their classification.

Integrins are heterodimeric entities, composed of *α* and *β* subunits, each 1TM proteins, which bind components of the extracellular matrix or counter‐receptors expressed on other cells. One class of integrin contains an inserted domain (I) in its *α* subunit, and if present (in *α*1, *α*2, *α*10, *α*11, *α*D, *α*E, *α*L, *α*M and *α*X), this I domain contains the ligand binding site. All *β* subunits possess a similar I‐like domain, which has the capacity to bind ligand, often recognising the RGD motif. The presence of an *α* subunit I domain precludes ligand binding through the *β* subunit. Integrins provide a link between ligand and the actin cytoskeleton (through typically short intracellular domains). Integrins bind several divalent cations, including a Mg^2+^ ion in the I or I‐like domain that is essential for ligand binding. Other cation binding sites may regulate integrin activity or stabilise the 3D structure. Integrins regulate the activity of particular protein kinases, including focal adhesion kinase and integrin‐linked kinase. Cellular activation regulates integrin ligand affinity via inside‐out signalling and ligand binding to integrins can regulate cellular activity via outside‐in signalling.

**Table:** 

Nomenclature	integrin *α*1*β*1	integrin *α*2*β*1	integrin *α*IIb*β*3	integrin *α*4*β*1
Subunits	integrin, beta 1 subunit (fibronectin receptor, beta polypeptide, antigen CD29 includes MDF2, MSK12), integrin, alpha 1 subunit	integrin, beta 1 subunit (fibronectin receptor, beta polypeptide, antigen CD29 includes MDF2, MSK12), integrin, alpha 2 subunit (CD49B, alpha 2 subunit of VLA‐2 receptor)	integrin, beta 3 subunit (platelet glycoprotein IIIa, antigen CD61), integrin, alpha IIb subunit (platelet glycoprotein IIb of IIb/IIIa complex, antigen CD41)	integrin, beta 1 subunit (fibronectin receptor, beta polypeptide, antigen CD29 includes MDF2, MSK12), integrin, alpha 4 subunit (antigen CD49D, alpha 4 subunit of VLA‐4 receptor)
Ligands	collagen, laminin	collagen, laminin, thrombospondin	fibrinogen (FGA FGB FGG, P02671 P02675 P02679), fibronectin (FN1, P02751), von Willebrand factor (VWF, P04275), vitronectin (VTN, P04004), thrombospondin	fibronectin (FN1, P02751), vascular cell adhesion protein 1 (VCAM1, P19320), osteopontin (SPP1, P10451), thrombospondin
Inhibitors	obtustatin (pIC_50_ 9.1) [118]	TCI15 (pIC_50_ 7.9) [129]	tirofiban (pIC_50_ 9.4) [182], G4120 (p*K*_i_ 8.4) [125, 204], GR 144053 (pIC_50_ 7.4) [40], eptifibatide (pIC_50_ 6.2–6.8) [160]	BIO1211 (pIC_50_ 8.3–9) [106], TCS2314
Antibodies	–	–	abciximab [34]	natalizumab [140]
Comments	–	–	–	LDV‐FITC is used as a probe at this receptor.

**Table:** 

Nomenclature	integrin *α*4*β*7	integrin *α*5*β*1	integrin *α*6*β*1	integrin *α*10*β*1
Subunits	integrin, alpha 4 subunit (antigen CD49D, alpha 4 subunit of VLA‐4 receptor), integrin, beta 7 subunit	integrin, beta 1 subunit (fibronectin receptor, beta polypeptide, antigen CD29 includes MDF2, MSK12), integrin, alpha 5 subunit (fibronectin receptor, alpha polypeptide)	integrin, beta 1 subunit (fibronectin receptor, beta polypeptide, antigen CD29 includes MDF2, MSK12), integrin, alpha 6 subunit	integrin, beta 1 subunit (fibronectin receptor, beta polypeptide, antigen CD29 includes MDF2, MSK12), integrin, alpha 10 subunit
Ligands	–	fibronectin (FN1, P02751)	laminin	collagen
Antibodies	vedolizumab (Antagonist) (pIC_50_ 8.3) [172]	–	–	–

**Table:** 

Nomenclature	integrin *α*11*β*1	integrin *α*E*β*7	integrin *α*L*β*2	integrin *α*V*β*3
Subunits	integrin, beta 1 subunit (fibronectin receptor, beta polypeptide, antigen CD29 includes MDF2, MSK12), integrin, alpha 11 subunit	integrin, alpha E subunit (antigen CD103, human mucosal lymphocyte antigen 1; alpha polypeptide), integrin, beta 7 subunit	integrin, beta 2 subunit (complement component 3 receptor 3 and 4 subunit), integrin, alpha L subunit (antigen CD11A (p180), lymphocyte function‐ associated antigen 1; alpha polypeptide)	integrin, beta 3 subunit (platelet glycoprotein IIIa, antigen CD61), integrin, alpha V subunit
Ligands	collagen	E‐cadherin	ICAM‐1 (ICAM1, P05362), ICAM‐2 (ICAM2, P13598)	vitronectin (VTN, P04004), fibronectin (FN1, P02751), fibrinogen (FGA FGB FGG, P02671 P02675 P02679), osteopontin (SPP1, P10451), von Willebrand factor (VWF, P04275), thrombospondin, tenascin
Activators	–	–	–	TP508 (p*K*_d_ 7.9) [36]
Inhibitors	–	–	A286982 (pIC_50_ 7.4–7.5) [109]	echistatin (pIC_50_ 11.7) [99], P11 (pIC_50_ 11.6) [99], cilengitide (pIC_50_ 8.5) [56]
Antibodies	–	–	–	etaracizumab (Binding) (p*K*_d_ 6.3) [199]

### Subunits

**Table:** 

Nomenclature	integrin, alpha 1 subunit	integrin, alpha 2 subunit (CD49B, alpha 2 subunit of VLA‐2 receptor)	integrin, alpha IIb subunit (platelet glycoprotein IIb of IIb/IIIa complex, antigen CD41)	integrin, alpha 3 subunit (antigen CD49C, alpha 3 subunit of VLA‐3 receptor)	integrin, alpha 4 subunit (antigen CD49D, alpha 4 subunit of VLA‐4 receptor)	integrin, alpha 5 subunit (fibronectin receptor, alpha polypeptide)
HGNC, UniProt	ITGA1, P56199	ITGA2, P08514	ITGA2B, P17301	ITGA3, P26006	ITGA4, P13612	ITGA5, P08648
Ligands	–	–	–	peptide ligand 2 (pIC_50_ 7.2) [203]	–	–
Antibodies	–	–	–	–	natalizumab [140]	–

**Table:** 

Nomenclature	integrin, alpha 6 subunit	integrin, alpha 7 subunit	integrin, alpha 8 subunit	integrin, alpha 9 subunit	integrin, alpha 10 subunit	integrin, alpha 11 subunit	integrin, alpha D subunit
HGNC, UniProt	ITGA6, P23229	ITGA7, Q13683	ITGA8, P53708	ITGA9, Q13797	ITGA10, O75578	ITGA11, Q9UKX5	ITGAD, Q13349

**Table:** 

Nomenclature	integrin, alpha E subunit (antigen CD103, human mucosal lymphocyte antigen 1; alpha polypeptide)	integrin, alpha L subunit (antigen CD11A (p180), lymphocyte function‐associated antigen 1; alpha polypeptide)	integrin, alpha M subunit (complement component 3 receptor 3 subunit)	integrin, alpha V subunit	integrin, alpha X subunit (complement component 3 receptor 4 subunit)
HGNC, UniProt	ITGAE, P38570	ITGAL, P20701	ITGAM, P11215	ITGAV, P06756	ITGAX, P20702
Antagonists	–	lifitegrast (Inhibition) [20, 207]	–	–	–
Antibodies	–	efalizumab (Binding) (p*K*_d_ 11.4) [96]	–	–	–

**Table:** 

Nomenclature	integrin, beta 2 subunit (complement component 3 receptor 3 and 4 subunit)	integrin, beta 3 subunit (platelet glycoprotein IIIa, antigen CD61)	integrin, beta 4 subunit	integrin, beta 5 subunit	integrin, beta 6 subunit	integrin, beta 7 subunit	integrin, beta 8 subunit	
HGNC, UniProt	ITGB1, P05556	ITGB2, P05107	ITGB3, P05106	ITGB4, P16144	ITGB5, P18084	ITGB6, P18564	ITGB7, P26010	ITGB8, P26012

### Comments: Integrin ligands

Collagen is the most abundant protein in metazoa, rich in glycine and proline residues, made up of cross‐linked triple helical structures, generated primarily by fibroblasts. Extensive post‐translational processing is conducted by prolyl and lysyl hydroxylases, as well as transglutaminases. Over 40 genes for collagen‐*α* subunits have been identified in the human genome. The collagen‐binding integrins *α*1*β*1, *α*2*β*1, *α*10*β*1 and *α*11*β*1 recognise a range of triple‐helical peptide motifs including GFOGER (O = hydroxyproline), a synthetic peptide derived from the primary sequence of collagen I (COL1A1(COL1A1, P02452)) and collagen II (COL2A1(COL2A1, P02458)).

Laminin is an extracellular glycoprotein composed of *α*, *β* and *γ* chains, for which five, four and three genes, respectively, are identified in the human genome. It binds to *α*1*β*1, *α*2*β*1, *α*3,*β*1, *α*7*β*1 and *α*6*β*4 integrins10.

fibrinogen(FGA FGB FGG, P02671 P02675 P02679) is a glycosylated hexamer composed of two *α*(FGA, P02671), two *β*(FGB, P02675) and two *γ*(FGG, P02679,) subunits, linked by disulphide bridges. It is found in plasma and alpha granules of platelets. It forms cross‐links between activated platelets mediating aggregation by binding *α*IIb*β*3; proteolysis by thrombin cleaves short peptides termed fibrinopeptides to generate fibrin, which polymerises as part of the blood coagulation cascade.

fibronectin(FN1, P02751) is a disulphide‐linked homodimer found as two major forms; a soluble dimeric form found in the plasma and a tissue version that is polymeric, which is secreted into the extracellular matrix by fibroblasts. Splice variation of the gene product (FN1, P02751) generates multiple isoforms.

vitronectin(VTN, P04004) is a serum glycoprotein and extracellular matrix protein which is found either as a monomer or, following proteolysis, a disulphide ‐linked dimer.

osteopontin(SPP1, P10451) forms an integral part of the mineralized matrix in bone, where it undergoes extensive post‐translation processing, including proteolysis and phosphorylation.

von Willebrand factor (VWF, P04275) is a glycoprotein synthesised in vascular endothelial cells as a disulphide‐linked homodimer, but multimerises further in plasma and is deposited on vessel wall collagen as a high molecular weight multimer. It is responsible for capturing platelets under arterial shear flow (via GPIb) and in thrombus propagation (via integrin *α*IIb*β*3).

### Further reading on Integrins

Clemetson, KJ. (2017) The origins of major platelet receptor nomenclature. Platelets 28: 40‐42 [PMID:27715379]

Hamidi, H et al. (2016) The complexity of integrins in cancer and new scopes for therapeutic targeting. Br J Cancer 115: 1017‐1023 [PMID:27685444]

Horton, ER et al. (2016) The integrin adhesome network at a glance. J Cell Sci 129: 4159‐4163 [PMID:27799358]

Ley, K et al. (2016) Integrin‐based therapeutics: biological basis, clinical use and new drugs. Nat Rev Drug Discov 15: 173‐83 [PMID:26822833]

Manninen, A et al. (2017) A proteomics view on integrin‐mediated adhesions. Proteomics 17: [PMID:27723259]

Park, YK et al. (2016) Integrins in synapse regulation. Nat Rev Neurosci 17: 745‐756 [PMID:27811927]

## Natriuretic peptide receptor family

### Overview

Natriuretic peptide receptors (NPRs, provisional nomenclature) are a family of homodimeric, catalytic receptors with a single TM domain and guanylyl cyclase (EC 4.6.1.2) activity on the intracellular domain of the protein sequence. Isoforms are activated by the peptide hormones atrial natriuretic peptide(NPPA, P01160), brain natriuretic peptide(NPPB, P16860) and C‐type natriuretic peptide (NPPC, P23582). Another family member is GC‐C, the receptor for guanylin(GUCA2A, Q02747) and uroguanylin(GUCA2B, Q16661). Family members have conserved ligand‐binding, catalytic (guanylyl cyclase) and regulatory domains with the exception of NPR‐C which has an extracellular binding domain homologous to that of other NPRs, but with a truncated intracellular domain which appears to couple, via the G_i/o_ family of G proteins, to activation of phospholipase C, inwardly‐rectifying potassium channels and inhibition of adenylyl cyclase activity [136].

**Table:** 

Nomenclature	Guanylyl cyclase‐A	Guanylyl cyclase‐B	Guanylyl cyclase‐C	natriuretic peptide receptor 3
HGNC, UniProt	NPR1, P16066	NPR2, P20594	GUCY2C, P25092	NPR3, P17342
Common abreviation	GC‐A	GC‐B	GC‐C	NPR‐C
Potency order	atrial natriuretic peptide (NPPA, P01160) ≥brain natriuretic peptide (NPPB, P16860) ≫C‐type natriuretic peptide (NPPC, P23582) [172]	C‐type natriuretic peptide (NPPC, P23582) ≫atrial natriuretic peptide (NPPA, P01160) ≫brain natriuretic peptide (NPPB, P16860) [172]	uroguanylin (GUCA2B, Q16661) >guanylin (GUCA2A, Q02747)	atrial natriuretic peptide (NPPA, P01160) >C‐type natriuretic peptide (NPPC, P23582) ≥brain natriuretic peptide (NPPB, P16860) [172]
Endogenous ligands	atrial natriuretic peptide (NPPA, P01160) [210] brain natriuretic peptide (NPPB, P16860) [210]	C‐type natriuretic peptide (NPPC, P23582) [172]	guanylin (GUCA2A, Q02747), uroguanylin (GUCA2B, Q16661)	osteocrin (OSTN, P61366) [148]
Selective agonists	Dendroaspis natriuretic peptide [211], sANP [210], cenderitide [212]	cenderitide [121], vosoritide [112]	linaclotide [18, 65], E. coli heat‐stable enterotoxin (STa) [18], plecanatide [190]	cANF4‐23 [132]
Selective antagonists	A‐71915 p*K*_i_ 9.2–9.5 [213], [Asu7,23']*β*‐ANP‐(7‐28) p*K*_i_ 7.5 [14], anantin [215, 216], HS142‐1 [217]	peptide P19 (p*K*d 7.8) [37], HS142‐1 [151], [Ser11](N‐CNP,C‐ANP)pBNP2‐15 [37], compound C10 [10]	–	AP811 (p*K*_i_ 9.3) [186], M372049 [73]
Labelled ligands	[^125^I]ANP (human) (Agonist)	[^125^I]CNP (human)	[^125^I]Sta (Agonist) [63]	[^125^I]ANP (human)

### Comments

The polysaccharide obtained from fermentation of Aureobasidium species, HS142‐1, acts as an antagonist at both GC‐A and GC‐B receptors [133]. GUCY2D(RetGC1, GC‐E, Q02846) and GUCY2F (RetGC2, GC‐F, P51841) are predominantly retinal guanylyl cyclase activities, which are inhibited by calcium ions acting through the guanylyl cyclase activating peptides GCAP1 (GUCA1A, 43080), GCAP2 (GUCA1B, Q9UMX6) and GCAP3 (GUCA1C, O95843) [76]. GC‐D and GC‐G are pseudogenes in man.

### Further reading on Natriuretic peptide receptor family

Blomain, ES et al. (2016) Guanylyl Cyclase C Hormone Axis at the Intersection of Obesity and Colorectal Cancer. Mol Pharmacol 90: 199‐204 [PMID:27251363]

Kuhn, M. (2016) Molecular Physiology of Membrane Guanylyl Cyclase Receptors. Physiol Rev 96: 751‐804 [PMID:27030537]

Santhekadur, PK et al. (2017) The multifaceted role of natriuretic peptides in metabolic syndrome. Biomed Pharmacother 92: 826‐835 [PMID:28599248]

Theilig, F et al. (2015) ANP‐induced signaling cascade and its implications in renal pathophysiology. Am J Physiol Renal Physiol 308: F1047‐55 [PMID:25651559]

Volpe, M et al. (2016) The natriuretic peptides system in the pathophysiology of heart failure: from molecular basis to treatment. Clin Sci (Lond) 130: 57‐77 [PMID:26637405]

## Pattern recognition receptors

### Overview

Pattern Recognition Receptors (PRRs, [173]) (nomenclature as agreed by NC‐IUPHAR sub‐committee on Pattern Recognition Receptors, [16]) participate in the innate immune response to microbial agents, the stimulation of which leads to activation of intracellular enzymes and regulation of gene transcription. PRRs express multiple leucine‐rich regions to bind a range of microbially‐derived ligands, termed PAMPs or pathogen‐associated molecular patterns, which includes peptides, carbohydrates, peptidoglycans, lipoproteins, lipopolysaccharides, and nucleic acids. PRRs include both cell‐surface and intracellular proteins. PRRs may be divided into signalling‐associated members, identified here, and endocytic members, the function of which appears to be to recognise particular microbial motifs for subsequent cell attachment, internalisation and destruction. Some are involved in inflammasome formation, and modulation IL‐1*β* cleavage and secretion, and others in the initiation of the type I interferon response.

PRRs included in the Guide To PHARMACOLOGY are:

Catalytic PRRs (see links below this overview)

Toll‐like receptors (TLRs)

Nucleotide‐binding oligomerization domain‐like receptors (NLRs, also known as NOD‐like receptors)

RIG‐I‐like receptors (RLRs)

### Non‐catalytic pattern recognition receptors

Absent in melanoma (AIM)‐like receptors (ALRs)

C‐type lectin‐like receptors (CLRs), and

Other pattern recognition receptors.

## Toll‐like receptor family

### Overview

Members of the toll‐like family of receptors (nomenclature recommended by the NC‐IUPHAR subcommittee on pattern recognition receptors, [16]) share significant homology with the interleukin‐1 receptor family and appear to require dimerization either as homo‐ or heterodimers for functional activity. Heterodimerization appears to influence the potency of ligand binding substantially (e.g. TLR1/2 and TLR2/6, [174, 175]). TLR1, TLR2, TLR4, TLR5, TLR6 and TLR11 are cell‐surface proteins, while other members are associated with intracellular organelles, signalling through the MyD88‐dependent pathways (with the exception of TLR3). As well as responding to exogenous infectious agents, it has been suggested that selected members of the family may be activated by endogenous ligands, such as hsp60(HSPD1, P10809) [161].

**Table:** 

Nomenclature	TLR1	TLR2	TLR3	TLR4	TLR5
HGNC, UniProt	TLR1, Q15399	TLR2, O60603	TLR3, O15455	TLR4, O00206	TLR5, O60602
Agonists	–	compound 13 [91], peptidoglycan [163, 205]	poly(I:C) [5]	LPS [150], paclitaxel [85] – Mouse	flagellin [67]
Selective antagonists	–	–	–	resatorvid [78]	–
Comments	Functions as a heterodimer with TLR2 in detection of triacylated lipoproteins. Activated by the synthetic analogue Pam3CSK4.	Functions as a heterodimer with either TLR1 or TLR6 in the detection of triacylated and diacylated lipopeptides respectively. TLR1/2 and 2/6 heterodimers can be activated by the synthetic lipopeptides Pam3CSK4 and Pam2CSK4 respectively. There is some debate in the field as to whether or not peptidoglycan is a direct agonist of TLR2, or whether the early studies reporting this contained contaminating lipoproteins.	Involved in endosomal detection of dsRNA; pro‐inflammatory.	eritoran (E5564) is a lipid A analogue, which has been described as a TLR4 antagonist [79]. TLR4 signals in conjunction with the co‐factor MD‐2 (LY96).	Involved in the detection of bacterial flagellin; pro‐inflammatory.

**Table:** 

Nomenclature	TLR6	TLR7	TLR8	TLR9	TLR10	TLR11
HGNC, UniProt	TLR6, Q9Y2C9	TLR7, Q9NYK1	TLR8, Q9NR97	TLR9, Q9NR96	TLR10, Q9BXR5	–
Agonists	–	imiquimod [70], loxoribine [68], resiquimod [70, 83]	resiquimod [70, 83]	–	–	–
Antagonists	–	hydroxychloroquine (pIC_50_ 5.6) [96]	–	hydroxychloroquine (pIC_50_ 7.1) [96]	–	–
Comments	Functions as a heterodimer with TLR2. Involved in the pro‐inflammatory response to diacylated bacterial lipopeptides.	Activated by imidazoquinoline derivatives and RNA oligoribonucleotides. Involved in endosomal detection of ssRNA; pro‐inflammatory.	Activated by imidazoquinoline derivatives and RNA oligoribonucleotides. Endosomal detection of ssRNA; pro‐inflammatory.	Toll‐like receptor 9 interacts with unmethylated CpG dinucleotides from bacterial DNA [71]. Activated by CpG rich DNA sequences; pro‐inflammatory.	TLR10 is the only pattern‐recognition receptor without known ligand specificity and biological function. Evidence suggests it plays a modulatory role with predominantly inhibitory (anti‐inflammatory) actions [145]. Murine TLR10 has a retroviral insertion that makes it non‐functional.	Found in mouse

### Further reading on Toll‐like receptor family

Bryant CE et al. (2015) International Union of Basic and Clinical Pharmacology. XCVI. Pattern recognition receptors in health and disease. Pharmacol Rev 67: 462‐504 [PMID:25829385]

Goulopoulou S et al. (2016) Toll‐like Receptors in the Vascular System: Sensing the Dangers Within. Pharmacol Rev 68: 142‐67 [PMID:26721702]

Micera A et al. (2016) Toll‐Like Receptors and Tissue Remodeling: The Pro/Cons Recent Findings. J Cell Physiol 231: 531‐44 [PMID:26248215]

Zhang Z et al. (2017) Towards a structural understanding of nucleic acid‐sensing Toll‐like receptors in the innate immune system. FEBS Lett [PMID:28686285]

Zinngrebe J et al. (2017) TLRs Go Linear ‐ On the Ubiquitin Edge. Trends Mol Med 23: 296‐309 [PMID:28325627]

## NOD‐like receptor family

### Overview

The nucleotide‐binding oligomerization domain, leucine‐rich repeat (NLR) family of receptors (nomenclature recommended by the NC‐IUPHAR subcommittee on pattern recognition receptors [16]) share a common domain organisation. This consists of an N‐terminal effector domain, a central nucleotide‐binding and oligomerization domain (NOD; also referred to as a NACHT domain), and C‐terminal leucine‐rich repeats (LRR) which have regulatory and ligand recognition functions. The type of effector domain has resulted in the division of NLR family members into two major sub‐families, NLRC and NLRP, along with three smaller sub‐families NLRA, NLRB and NLRX [177]. NLRC members express an N‐terminal caspase recruitment domain (CARD) and NLRP members an N‐terminal Pyrin domain (PYD).

Upon activation the NLRC family members NOD1 (NLRC1) and NOD2 (NLRC2) recruit a serine/threonine kinase RIPK2(receptor interacting serine/threonine kinase 2, O43353, also known as CARD3, CARDIAK, RICK, RIP2) leading to signalling through NF*κ*B and MAP kinase. Activation of NLRC4 (previously known as IPAF) and members of the NLRP3 family, including NLRP1 and NLRP3, leads to formation of a large multiprotein complex known as the inflammasome. In addition to NLR proteins other key members of the inflammasome include the adaptor protein ASC (apoptosis‐associated speck‐like protein containing a CARD, also known as PYCARD, CARD5, TMS1, Q9ULZ3) and inflammatory caspases. The inflammasome activates the pro‐inflammatory cytokines IL‐1*β*(IL1B, P01584) and IL‐18(IL18, Q14116) [16, 32].

**Table:** 

Nomenclature	nucleotide binding oligomerization domain containing 1	nucleotide binding oligomerization domain containing 2	NLRC3	NLRC4	NLRC5	NLRX1	CIITA
HGNC, UniProt	NOD1, Q9Y239	NOD2, Q9HC29	NLRC3, Q7RTR2	NLRC4, Q9NPP4	NLRC5, Q86WI3	NLRX1, Q86UT6	CIITA, P33076
Common abreviation	NOD1	NOD2	–	–	–	–	–
Agonists	meso‐DAP	muramyl dipeptide	–	–	–	–	–
Comments	–	NOD2 has also been reported to be activated by ssRNA [158] although this has not been widely reproduced.	–	NLRC4 forms an inflammasome in conjunction with the NAIP proteins and responds to bacterial flagellin and type III secretion system rod proteins.	–	–	–

**Table:** 

Nomenclature	NLRP1	NLRP2	NLRP3	NLRP4	NLRP5	NLRP6	NLRP7
HGNC, UniProt	NLRP1, Q9C000	NLRP2, Q9NX02	NLRP3, Q96P20	NLRP4, Q96MN2	NLRP5, P59047	NLRP6, P59044	NLRP7, Q8WX94
Inhibitors	–	–	MCC950 (pIC_50_>8) [25]	–	–	–	–
Agonists	muramyl dipeptide	–	–	–	–	–	–
Comments	NLRP1 has 3 murine orthologues which lack the N‐terminal Pyrin domain. Murine NLRP1b (ENSMUSG00000070390) is the best characterised, responding to Anthrax Lethal Toxin.	Along with NLRP7, NLRP2 is the product of a primate‐specific gene duplication.	Multiple virus particles have been shown to act as agonists, including Sendai and influenza. NLRP3 has been shown to be activated following disruption of cellular haemostasis by a wide‐variety of exogenous and endogenous molecules. The identity of the precise agonist that interacts with NLRP3 remains enigmatic.	Expanded in the mouse resulting in 7 orthologues.	–	–	Absent in mouse. Along with NLRP2 the product of a primate‐specific gene duplication.

**Table:** 

Nomenclature	NLRP8	NLRP9	NLRP10	NLRP11	NLRP12	NLRP13	NLRP14
HGNC, UniProt	NLRP8, Q86W28	NLRP9, Q7RTR0	NLRP10, Q86W26	NLRP11, P59045	NLRP12, P59046	NLRP13, Q86W25	NLRP14, Q86W24
Comments	Absent in mouse	This receptor has three murine orthologues.	–	Absent in mouse	–	Absent in mouse	–

### Comments

NLRP3 has also been reported to respond to host‐derived products, known as danger‐associated molecular patterns, or DAMPs, including uric acid [141], ATP, L‐glucose, hyaluronan and amyloid *β*(APP, P05067) [161].

Loss‐of‐function mutations of NLRP3 are associated with cold autoinflammatory and Muckle‐Wells syndromes.

This family also includes NLR family, apoptosis inhibitory protein (NAIP, Q13075) which can be found in the 'Inhibitors of apoptosis (IAP) protein family' in the Other protein targets section of the Guide.

### Further reading on NOD‐like receptor family

Bryant CE et al. (2015) International Union of Basic and Clinical Pharmacology. XCVI. Pattern recognition receptors in health and disease. Pharmacol Rev 67: 462‐504 [PMID:25829385]

Kong X et al. (2017) The function of NOD‐like receptors in central nervous system diseases. J Neurosci Res 95: 1565‐1573 [PMID:28029680]

Motta V et al. (2015) NOD‐like receptors: versatile cytosolic sentinels. Physiol Rev 95: 149‐78 [PMID:25540141]

## Receptor tyrosine kinases (RTKs)

### Overview

Receptor tyrosine kinases (RTKs), a family of cell‐surface receptors, which transduce signals to polypeptide and protein hormones, cytokines and growth factors are key regulators of critical cellular processes, such as proliferation and differentiation, cell survival and metabolism, cell migration and cell cycle control [11, 57, 184]. In the human genome, 58 RTKs have been identified, which fall into 20 families [100].

All RTKs display an extracellular ligand binding domain, a single transmembrane helix, a cytoplasmic region containing the protein tyrosine kinase activity (occasionally split into two domains by an insertion, termed the kinase insertion), with juxta‐membrane and C‐terminal regulatory regions. Agonist binding to the extracellular domain evokes dimerization, and sometimes oligomerization, of RTKs (a small subset of RTKs forms multimers even in the absence of activating ligand). This leads to autophosphorylation in the tyrosine kinase domain in a trans orientation, serving as a site of assembly of protein complexes and stimulation of multiple signal transduction pathways, including phospholipase C‐*γ*, mitogen‐activated protein kinases and phosphatidylinositol 3‐kinase [184].

RTKs are of widespread interest not only through physiological functions, but also as drug targets in many types of cancer and other disease states. Many diseases result from genetic changes or abnormalities that either alter the activity, abundance, cellular distribution and/or regulation of RTKs. Therefore, drugs that modify the dysregulated functions of these RTKs have been developed which fall into two categories. One group is often described as ‘biologicals’, which block the activation of RTKs directly or by chelating the cognate ligands, while the second are small molecules designed to inhibit the tyrosine kinase activity directly.

## Type I RTKs: ErbB (epidermal growth factor) receptor family

### Overview

ErbB family receptors are Class I receptor tyrosine kinases [57]. ERBB2 (also known as HER‐2 or NEU) appears to act as an essential partner for the other members of the family without itself being activated by a cognate ligand [58]. Ligands of the ErbB family of receptors are peptides, many of which are generated by proteolytic cleavage of cell‐surface proteins. HER/ErbB is the viral counterpart to the receptor tyrosine kinase EGFR. All family members heterodimerize with each other to activate downstream signalling pathways and are aberrantly expressed in many cancers, particularly forms of breast cancer and lung cancer. Mutations in the EGFR are responsible for acquired resistance to tyrosine kinase inhibitor chemotherapeutics.

**Table:** 

Nomenclature	epidermal growth factor receptor	erb‐b2 receptor tyrosine kinase 2	erb‐b2 receptor tyrosine kinase 3	erb‐b2 receptor tyrosine kinase 4
HGNC, UniProt	EGFR, P00533	ERBB2, P04626	ERBB3, P21860	ERBB4, Q15303
EC number	2.7.10.1	2.7.10.1	2.7.10.1	2.7.10.1
Common abreviation	EGFR	HER2	HER3	HER4
Endogenous ligands	EGF (EGF, P01133), HB‐EGF (HBEGF, Q99075), TGF*α* (TGFA, P01135), amphiregulin (AREG, P15514), betacellulin (BTC, P35070), epigen (EPGN, Q6UW88), epiregulin (EREG, O14944)	–	neuregulin‐1 (NRG1, Q02297), neuregulin‐2 (NRG2, O14511)	HB‐EGF (HBEGF, Q99075), betacellulin (BTC, P35070), epiregulin (EREG, O14944), neuregulin‐1 (NRG1, Q02297), neuregulin‐2 (NRG2, O14511), neuregulin‐3 (NRG3, P56975), neuregulin‐4 (NRG4, Q8WWG1)
Inhibitors	canertinib (p*K*_d_ 9.7) [33], afatinib (p*K*_d_ 9.6) [33], tesevatinib (pIC_50_ 9.5) [63], afatinib (pIC_50_ 8–9.3) [36, 119], erlotinib (p*K*_d_ 9.2) [33], gefitinib (p*K*_d_ 9) [33],	poziotinib (pIC_50_ 8.3) [139], neratinib (p*K*_d_ 8.2) [33], lapatinib (p*K*_d_ 8.1) [33], CP‐724714 (pIC_50_ 7.9) [60], tesevatinib (pIC_50_ 7.8) [63], BMS‐690514 (pIC_50_ 7.7) [117]	–	poziotinib (pIC_50_ 7.6) [139]
Antibodies	necitumumab (Binding) (p*K*_d_ 9.5) [128], cetuximab (Binding) (p*K*_d_ 9.4) [66]	pertuzumab (Inhibition) (pIC_50_>8) [100], trastuzumab (Inhibition)	–	–

### Comments

[^125^I]EGF (human) has been used to label the ErbB1 EGF receptor. The extracellular domain of ErbB2 can be targetted by the antibodies trastuzumab and pertuzumab to inhibit ErbB family action. The intracellular ATP‐binding site of the tyrosine kinase domain can be inhibited by GW583340(7.9–8.0, [50]), gefitinib, erlotinib and tyrphostins AG879 and AG1478.

### Further reading on Type I RTKs: ErbB (epidermal growth factor) receptor family

Kobayashi Y et al. (2016) Not all EGFR mutations in lung cancer are created equal: Perspectives for individualized treatment strategy. Cancer Sci. [PMID:27323238]

## Type II RTKs: Insulin receptor family

### Overview

The circulating peptide hormones insulin(INS, P01308) and the related insulin‐like growth factors (IGF) activate Class II receptor tyrosine kinases [57], to evoke cellular responses, mediated through multiple intracellular adaptor proteins. Exceptionally amongst the catalytic receptors, the functional receptor in the insulin receptor family is derived from a single gene product, cleaved post‐translationally into two peptides, which then cross‐link via disulphide bridges to form a heterotetramer. Intriguingly, the endogenous peptide ligands are formed in a parallel fashion with post‐translational processing producing a heterodimer linked by disulphide bridges. Signalling through the receptors is mediated through a rapid autophosphorylation event at intracellular tyrosine residues, followed by recruitment of multiple adaptor proteins, notably IRS1 (P35568), IRS2 (Q9Y4H2), SHC1 (P29353), GRB2 (P62993) and SOS1(Q07889).

Serum levels of free IGFs are kept low by the action of IGF binding proteins (IGFBP1‐5, P08833, P18065, P17936, P22692, P24593), which sequester the IGFs; overexpression of IGFBPs may induce apoptosis, while IGFBP levels are also altered in some cancers.

**Table:** 

Nomenclature	Insulin receptor	Insulin‐like growth factor I receptor	Insulin receptor‐related receptor
HGNC, UniProt	INSR, P06213	IGF1R, P08069	INSRR, P14616
EC number	2.7.10.1	2.7.10.1	2.7.10.1
Common abreviation	InsR	IGF1R	IRR
Inhibitors	–	BMS‐754807 (pIC_50_ 8.7) [198], GSK‐1838705A (pIC_50_ 8.7) [159], GSK‐1838705A (p*K*_d_ 8.1) [33], PQ401 (pIC_50_>6) [47], AG 1024 (pIC_50_ 4.7) [153]	–
Selective inhibitors	–	NVP‐AEW541 (pIC_50_ 9.4) [49]	–
Endogenous agonists	insulin (INS, P01308)	insulin‐like growth factor 1 (IGF1, P05019), insulin‐like growth factor 2 (IGF2, P01344)	–

### Comments

There is evidence for low potency binding and activation of insulin receptors by IGF1. IGF2 also binds and activates the cation‐independent mannose 6‐phosphate receptor (also known as the insulin‐like growth factor 2 receptor; IGF2R; P11717), which lacks classical signalling capacity and appears to subserve a trafficking role [115]. INSRR, which has a much more discrete localization, being predominant in the kidney [93], currently lacks a cognate ligand or evidence for functional impact.

Antibodies targetting IGF1, IGF2 and the extracellular portion of the IGF1 receptor are in clinical trials.

PQ401 inhibits the insulin‐like growth factor receptor [5], while BMS‐536924 inhibits both the insulin receptor and the insulin‐like growth factor receptor [197].

## Type III RTKs: PDGFR, CSFR, Kit, FLT3 receptor family

### Overview

Type III RTKs include PDGFR, CSF‐1R (Ems), Kit and FLT3, which function as homo‐ or heterodimers. Endogenous ligands of PDGF receptors are homo‐ or heterodimeric: PDGFA, PDGFB, VEGFE and PDGFD(PDGFD, Q9GZP0) combine as homo‐ or heterodimers to activate homo‐ or heterodimeric PDGF receptors. SCF is a dimeric ligand for KIT. Ligands for CSF1R are either monomeric or dimeric glycoproteins, while the endogenous agonist for FLT3 is a homodimer.

**Table:** 

Nomenclature	platelet derived growth factor receptor alpha	platelet derived growth factor receptor beta	KIT proto‐oncogene receptor tyrosine kinase
HGNC, UniProt	PDGFRA, P16234	PDGFRB, P09619	KIT, P10721
EC number	2.7.10.1	2.7.10.1	2.7.10.1
Common abreviation	PDGFR*α*	PDGFR*β*	Kit
Endogenous ligands	PDGF	PDGF	–
Inhibitors	PP121 (pIC_50_ 8.7) [4], crenolanib (p*K*_d_ 8.7) [69], ENMD‐2076 (pIC_50_ 7.2) [149]	crenolanib (p*K*_d_ 8.5) [69], SU‐14813 (pIC_50_ 8.4) [147], famitinib (pIC_50_ 8.4) [21], sunitinib (pIC_50_ 8.2) [89], sunitinib (p*K*_i_ 8.1) [127]	sunitinib (p*K*_d_ 9.4) [33], famitinib (pIC_50_ 8.7) [21], masitinib (p*K*_d_ 8.1) [33], SU‐14813 (pIC_50_ 7.8) [147], AKN‐028 (pIC_50_ 7.5) [42], sorafenib (pIC_50_ 7.2) [196]
Selective inhibitors	CP‐673451 (pIC_50_ 8) [156]	CP‐673451 (pIC_50_ 9) [156]	–
Endogenous agonists	–	–	stem cell factor (KITLG, P21583) [183]

**Table:** 

Nomenclature	colony stimulating factor 1 receptor	fms related tyrosine kinase 3
HGNC, UniProt	CSF1R, P07333	FLT3, P36888
EC number	2.7.10.1	2.7.10.1
Common abreviation	CSFR	FLT3
Endogenous ligands	G‐CSF (CSF3, P09919), GM‐CSF (CSF2, P04141), M‐CSF (CSF1, P09603)	Fms‐related tyrosine kinase 3 ligand (FLT3LG, P49771)
Inhibitors	JNJ‐28312141 (pIC_50_ 9.2) [116], Ki‐20227 (p*K*_d_ 9.1) [33], Ki‐20227 (pIC_50_ 8.7) [143], GW‐2580 (p*K*_d_ 8.7) [33], JNJ‐28312141 (p*K*_d_ 8.5) [33]	AC710 (p*K*_d_ 9.3) [108], linifanib (p*K*_d_ 9.2) [33], dovitinib (p*K*_d_ 9.2) [33], crenolanib (p*K*_d_ 9.1) [69], AST‐487 (p*K*_d_ 9.1) [33], ENMD‐2076 (pIC_50_ 8.5) [149], tandutinib (p*K*_d_ 8.5) [33]
Selective inhibitors	GW‐2580 (pIC_50_ 7.2) [27]	G749 (pIC_50_ 9.4) [97]
Comments	Upregulation of CSF1R expression is associated with migroglial activation and immune pathology in Alzhermer's disease (AD) [67, 61]. Pharmacological inhibition of CSF1R with GW‐2580 reduces microglial proliferation and prevents disease progression in a mouse model of AD, but this does not correlate with amyloid‐*β* plaque numbers [144].	5'‐fluoroindirubinoxime has been described as a selective FLT3 inhibitor [22].

### Comments

Various small molecular inhibitors of type III RTKs have been described, including imatinib and nilotinib (targetting PDGFR, KIT and CSF1R); midostaurin and AC220 (quizartinib; FLT3), as well as pan‐type III RTK inhibitors such as sunitinib and sorafenib[148]; 5'‐fluoroindirubinoxime has been described as a selective FLT3 inhibitor [1].

## Type IV RTKs: VEGF (vascular endothelial growth factor) receptor family

### Overview

VEGF receptors are homo‐ and heterodimeric proteins, which are characterized by seven Ig‐like loops in their extracellular domains and a split kinase domain in the cytoplasmic region. They are key regulators of angiogenesis and lymphangiogenesis; as such, they have been the focus of drug discovery for conditions such as metastatic cancer. Splice variants of VEGFR1 and VEGFR2 generate truncated proteins limited to the extracellular domains, capable of homodimerisation and binding VEGF ligands as a soluble, non‐signalling entity. Ligands at VEGF receptors are typically homodimeric. VEGFA(VEGFA, P15692) is able to activate VEGFR1 homodimers, VEGFR1/2 heterodimers and VEGFR2/3 heterodimers. VEGFB(VEGFB, P49765) and placental growth factor (PGF, P49763) activate VEGFR1 homodimers, while VEGFC(VEGFC, P49767) and VEGFD(VEGFD, O43915) activate VEGFR2/3 heterodimers and VEGFR3 homodimers, and, following proteolysis, VEGFR2 homodimers.

**Table:** 

Nomenclature	fms related tyrosine kinase 1	kinase insert domain receptor	fms related tyrosine kinase 4
HGNC, UniProt	FLT1, P17948	KDR, P35968	FLT4, P35916
EC number	2.7.10.1	2.7.10.1	2.7.10.1
Common abreviation	VEGFR‐1	VEGFR‐2	VEGFR‐3
Endogenous ligands	VEGFA (VEGFA, P15692), VEGFB (VEGFB, P49765)	VEGFA (VEGFA, P15692), VEGFC (VEGFC, P49767), VEGFE (PDGFC, Q9NRA1)	VEGFC (VEGFC, P49767), VEGFD (VEGFD, O43915), VEGFE (PDGFC, Q9NRA1)
Inhibitors	SU‐14813 (pIC_50_ 8.7) [147], CEP‐11981 (pIC_50_ 8.5) [75], semaxanib (pIC_50_ 8.1) [12]	axitinib (pIC_50_ 9.6) [98], cabozantinib (pIC_50_ 9.5) [200], foretinib (pIC_50_ 8.2–9.1) [137], cediranib (p*K*_d_ 9) [33], tesevatinib (pIC_50_ 8.8) [51], SU‐14813 (p*K*_d_ 8.6) [33], motesanib (p*K*_d_ 8.6) [33], famitinib (pIC_50_ 8.3) [21], axitinib (p*K*_d_ 8.2) [33]	tesevatinib (pIC_50_ 8.1) [51], sunitinib (pIC_50_ 8.1) [86], nintedanib (pIC_50_ 7.9) [72]
Sub/family‐selective inhibitors	pazopanib (pIC_50_ 8) [66]	pazopanib (p*K*_d_ 7.8) [33], pazopanib (pIC_50_ 7.5) [79]	pazopanib (pIC_50_ 7.3) [66]
Antibodies	–	ramucirumab (Antagonist) (pIC_50_ 9) [113]	–

### Comments

The VEGFR, as well as VEGF ligands, have been targeted by antibodies and tyrosine kinase inhibitors. DMH4 [45], Ki8751 [92] and ZM323881, a novel inhibitor of vascular endothelial growth factor‐receptor‐2 tyrosine kinase activity [193] are described as VEGFR2‐selective tyrosine kinase inhibitors. Bevacizumab is a monoclonal antibody directed against VEGF‐A, used clinically for the treatment of certain metastatic cancers; an antibody fragment has been used for wet age‐related macular degeneration.

## Type V RTKs: FGF (fibroblast growth factor) receptor family

### Overview

Fibroblast growth factor (FGF) family receptors act as homo‐ and heterodimers, and are characterized by Ig‐like loops in the extracellular domain, in which disulphide bridges may form across protein partners to allow the formation of covalent dimers which may be constitutively active. FGF receptors have been implicated in achondroplasia, angiogenesis and numerous congenital disorders. At least 22 members of the FGF gene family have been identified in the human genome [8]. Within this group, subfamilies of FGF may be divided into canonical, intracellular and hormone‐like FGFs. FGF1‐FGF10 have been identified to act through FGF receptors, while FGF11‐14 appear to signal through intracellular targets. Other family members are less well characterized [192].

**Table:** 

Nomenclature	fibroblast growth factor receptor 1	fibroblast growth factor receptor 2	fibroblast growth factor receptor 3	fibroblast growth factor receptor 4
HGNC, UniProt	FGFR1, P11362	FGFR2, P21802	FGFR3, P22607	FGFR4, P22455
EC number	2.7.10.1	2.7.10.1	2.7.10.1	2.7.10.1
Common abreviation	FGFR1	FGFR2	FGFR3	FGFR4
Endogenous ligands	FGF‐1 (FGF1, P05230), FGF‐2 (FGF2, P09038), FGF‐4 (FGF4, P08620) >FGF‐5 (FGF5, P12034), FGF‐6 (FGF6, P10767) [146]	FGF‐1 (FGF1, P05230) >FGF‐4 (FGF4, P08620), FGF‐7 (FGF7, P21781), FGF‐9 (FGF9, P31371) >FGF‐2 (FGF2, P09038), FGF‐6 (FGF6, P10767) [146]	FGF‐1 (FGF1, P05230), FGF‐2 (FGF2, P09038), FGF‐9 (FGF9, P31371) >FGF‐4 (FGF4, P08620), FGF‐8 (FGF8, P55075) [146]	FGF‐1 (FGF1, P05230), FGF‐2 (FGF2, P09038), FGF‐4 (FGF4, P08620), FGF‐9 (FGF9, P31371) >FGF‐6 (FGF6, P10767), FGF‐8 (FGF8, P55075) [146]
Sub/family‐selective inhibitors	LY2874455 (pIC_50_ 8.6) [205]	LY2874455 (pIC_50_ 8.6) [205]	LY2874455 (pIC_50_ 8.2) [205]	LY2874455 (pIC_50_ 8.2) [205]
Selective inhibitors	–	–	–	BLU9931 (pIC_50_ 8.5) [75]
Agonists	–	palifermin	–	–

### Comments

Splice variation of the receptors can influence agonist responses. FGFRL1(Q8N441) is a truncated kinase‐null analogue.

Various antibodies and tyrosine kinase inhibitors have been developed against FGF receptors [105, 209]. PD161570 is an FGFR tyrosine kinase inhibitor [8], while PD173074 has been described to inhibit FGFR1 and FGFR3 [168].

## Type VI RTKs: PTK7/CCK4

### Overview

The PTK7 receptor is associated with polarization of epithelial cells and the development of neural structures. Sequence analysis suggests that the gene product is catalytically inactive as a protein kinase, although there is evidence for a role in Wnt signalling [152].

**Table:** 

Nomenclature	protein tyrosine kinase 7 (inactive)
HGNC, UniProt	PTK7, Q13308
EC number	2.7.10.1
Common abreviation	CCK4

### Comments

Thus far, no selective PTK7 inhibitors have been described.

## Type VII RTKs: Neurotrophin receptor/Trk family

### Overview

The neurotrophin receptor family of RTKs include trkA, trkB and trkC (tropomyosin‐related kinase) receptors, which respond to NGF, BDNF and neurotrophin‐3, respectively. They are associated primarily with proliferative and migration effects in neural systems. Various isoforms of neurotrophin receptors exist, including truncated forms of trkB and trkC, which lack catalytic domains. p75 (TNFRSF16, also known as nerve growth factor receptor), which has homologies with tumour necrosis factor receptors, lacks a tyrosine kinase domain, but can signal via ceramide release and nuclear factor *κ*B (NF‐*κ*B) activation. Both trkA and trkB contain two leucine‐rich regions and can exist in monomeric or dimeric forms.

**Table:** 

Nomenclature	neurotrophic receptor tyrosine kinase 1	neurotrophic receptor tyrosine kinase 2	neurotrophic receptor tyrosine kinase 3
HGNC, UniProt	NTRK1, P04629	NTRK2, Q16620	NTRK3, Q16288
EC number	2.7.10.1	2.7.10.1	2.7.10.1
Common abreviation	trkA	trkB	trkC
Endogenous ligands	NGF (NGF, P01138) >neurotrophin‐3 (NTF3, P20783)	BDNF (BDNF, P23560), neurotrophin‐4 (NTF4, P34130) >neurotrophin‐3 (NTF3, P20783)	neurotrophin‐3 (NTF3, P20783)
Inhibitors	compound 2c (pIC_50_ 8.9) [189], milciclib (pIC_50_ 7.3) [14]	–	–
Sub/family‐selective inhibitors	AZD1332 (pIC_50_>8.3) [9], GNF‐5837 (pIC_50_ 8) [2]	AZD1332 (pIC_50_>8.3) [9], GNF‐5837 (pIC_50_ 8.1) [2]	AZD1332 (pIC_50_>8.3) [9], GNF‐5837 (pIC_50_ 8.1) [2]

### Comments

[^125^I]NGF (human) and [^125^I]BDNF (human) have been used to label the trkA and trkB receptor, respectively. p75 influences the binding of NGF(NGF, P01138) and neurotrophin‐3(NTF3, P20783) to trkA. The ligand selectivity of p75 appears to be dependent on the cell type; for example, in sympathetic neurones, it binds neurotrophin‐3(NTF3, P20783) with comparable affinity to trkC [35].

Small molecule agonists of trkB have been described, including LM22A4 [124], while ANA12 has been described as a non‐competitive antagonist of BDNF binding to trkB [20]. GNF5837 is a family‐selective tyrosine kinase inhibitor [2], while the tyrosine kinase activity of the trkA receptor can be inhibited by GW441756(pIC_50_= 8.7, [198]) and tyrphostin AG879[162].

## Type VIII RTKs: ROR family

### Overview

Members of the ROR family appear to be activated by ligands complexing with other cell‐surface proteins. Thus, ROR1 and ROR2 appear to be activated by Wnt‐5a(WNT5A, P41221) binding to a Frizzled receptor thereby forming a cell‐surface multiprotein complex [59].

**Table:** 

Nomenclature	receptor tyrosine kinase like orphan receptor 1	receptor tyrosine kinase like orphan receptor 2
HGNC, UniProt	ROR1, Q01973	ROR2, Q01974
EC number	2.7.10.1	2.7.10.1
Common abreviation	ROR1	ROR2

## Type IX RTKs: MuSK

### Overview

The muscle‐specific kinase MuSK is associated with the formation and organisation of the neuromuscular junction from the skeletal muscle side. Agrin(AGRN, O00468) forms a complex with low‐density lipoprotein receptor‐related protein 4 (LRP4, O75096) to activate MuSK [87].

**Table:** 

Nomenclature	muscle associated receptor tyrosine kinase
HGNC, UniProt	MUSK, O15146
EC number	2.7.10.1
Common abreviation	MuSK

### Comments

Thus far, no selective MuSK inhibitors have been described.

## Type X RTKs: HGF (hepatocyte growth factor) receptor family

### Overview

HGF receptors regulate maturation of the liver in the embryo, as well as having roles in the adult, for example, in the innate immune system. HGF is synthesized as a single gene product, which is post‐translationally processed to yield a heterodimer linked by a disulphide bridge. The maturation of HGF is enhanced by a serine protease, HGF activating complex, and inhibited by HGF‐inhibitor 1 (SPINT1, O43278), a serine protease inhibitor. MST1, the ligand of RON, is two disulphide‐linked peptide chains generated by proteolysis of a single gene product.

**Table:** 

Nomenclature	MET proto‐oncogene, receptor tyrosine kinase	macrophage stimulating 1 receptor
HGNC, UniProt	MET, P08581	MST1R, Q04912
EC number	2.7.10.1	2.7.10.1
Common abreviation	MET	Ron
Endogenous ligands	hepatocyte growth factor (HGF, P14210)	macrophage stimulating protein 1 (MST1, P09603)
Inhibitors	capmatinib (pIC_50_ 9.9) [111], SGX‐523 (p*K*_d_ 9.7) [33], PHA‐665752 (p*K*_d_ 9.6) [33], foretinib (pIC_50_ 9.3–9.4) [104, 137], cabozantinib (pIC_50_ 8.9) [200]	BMS‐777607 (pIC_50_ 8.7) [162]
Selective inhibitors	SGX‐523 (pIC_50_ 8.4) [17]	–

### Comments

PF04217903 is a selective Met tyrosine kinase inhibitor [29]. SU11274 is an inhibitor of the HGF receptor [185], with the possibility of further targets [5].

## Type XI RTKs: TAM (TYRO3‐, AXL‐ and MER‐TK) receptor family

### Overview

Members of this RTK family represented a novel structural motif, when sequenced. The ligands for this family, growth arrest specific protein 6(GAS6, Q14393) and protein S (PROS1, P07225), are secreted plasma proteins which undergo vitamin K‐dependent post‐translational modifications generating carboxyglutamate‐rich domains which are able to bind to negatively‐charged surfaces of apoptotic cells.

**Table:** 

Nomenclature	AXL receptor tyrosine kinase	TYRO3 protein tyrosine kinase	MER proto‐oncogene, tyrosine kinase
HGNC, UniProt	AXL, P30530	TYRO3, Q06418	MERTK, Q12866
EC number	2.7.10.1	2.7.10.1	2.7.10.1
Common abreviation	Axl	Tyro3	Mer
Endogenous ligands	growth arrest specific protein 6 (GAS6, Q14393) [138], protein S (PROS1, P07225) [171]	growth arrest specific protein 6 (GAS6, Q14393) [138], protein S (PROS1, P07225) [171]	growth arrest specific protein 6 (GAS6, Q14393) [138]

### Comments

AXL tyrosine kinase inhibitors have been described [131].

## Type XII RTKs: TIE family of angiopoietin receptors

### Overview

The TIE family were initially associated with formation of blood vessels. Endogenous ligands are angiopoietin‐1(ANGPT1, Q15389), angiopoietin‐2(ANGPT2, O15123), and angiopoietin‐4(ANGPT4, Q9Y264). Angiopoietin‐2(ANGPT2, O15123) appears to act as an endogenous antagonist of angiopoietin‐1 function.

**Table:** 

Nomenclature	tyrosine kinase with immunoglobulin like and EGF like domains 1	TEK receptor tyrosine kinase
HGNC, UniProt	TIE1, P35590	TEK, Q02763
EC number	2.7.10.1	2.7.10.1
Common abreviation	TIE1	TIE2
Endogenous ligands	–	angiopoietin‐1 (ANGPT1, Q15389), angiopoietin‐4 (ANGPT4, Q9Y264)

## Type XIII RTKs: Ephrin receptor family

### Overview

Ephrin receptors are a family of 15 RTKs (the largest family of RTKs) with two identified subfamilies (EphA and EphB), which have a role in the regulation of neuronal development, cell migration, patterning and angiogenesis. Their ligands are membrane‐associated proteins, thought to be glycosylphosphatidylinositol‐linked for EphA (ephrin‐A1(EFNA1, P20827), ephrin‐A2(EFNA2, O43921), ephrin‐A3(EFNA3, P52797), ephrin‐A4(EFNA4, P52798) and ephrin‐A5(EFNA5, P52803)) and 1TM proteins for Ephrin B (ENSFM00250000002014: ephrin‐B1(EFNB1, P98172), ephrin‐B2(EFNB2, P52799) and ephrin‐B3(EFNB3, Q15768)), although the relationship between ligands and receptors has been incompletely defined.

**Table:** 

Nomenclature	EPH receptor A1	EPH receptor A2	EPH receptor A3	EPH receptor A4	EPH receptor A5	EPH receptor A6	EPH receptor A7
HGNC, UniProt	EPHA1, P21709	EPHA2, P29317	EPHA3, P29320	EPHA4, P54764	EPHA5, P54756	EPHA6, Q9UF33	EPHA7, Q15375
EC number	2.7.10.1	2.7.10.1	2.7.10.1	2.7.10.1	2.7.10.1	2.7.10.1	2.7.10.1
Common abreviation	EphA1	EphA2	EphA3	EphA4	EphA5	EphA6	EphA7

**Table:** 

Nomenclature	EPH receptor A8	EPH receptor A10	EPH receptor B1	EPH receptor B2	EPH receptor B3	EPH receptor B4	EPH receptor B6
HGNC, UniProt	EPHA8, P29322	EPHA10, Q5JZY3	EPHB1, P54762	EPHB2, P29323	EPHB3, P54753	EPHB4, P54760	EPHB6, O15197
EC number	2.7.10.1	2.7.10.1	2.7.10.1	2.7.10.1	2.7.10.1	2.7.10.1	2.7.10.1
Common abreviation	EphA8	EphA10	EphB1	EphB2	EphB3	EphB4	EphB6
Inhibitors	–	–	compound 66 (pIC_50_ 9) [95]	–	–	tesevatinib (pIC_50_ 8.9) [51]	–

## Type XIV RTKs: RET

### Overview

Ret proto‐oncogene (Rearranged during transfection) is a transmembrane tyrosine kinase enzyme which is employed as a signalling partner for members of the GDNF family receptors. Ligand‐activated GFR appears to recruit Ret as a dimer, leading to activation of further intracellular signalling pathways. Ret appears to be involved in neural crest development, while mutations may be involved in multiple endocrine neoplasia, Hirschsprung's disease, and medullary thyroid carcinoma.

**Table:** 

Nomenclature	ret proto‐oncogene
HGNC, UniProt	RET, P07949
EC number	2.7.10.1
Common abreviation	Ret
Inhibitors	tamatinib (pIC_50_ 8.3) [23], vandetanib (p*K*_d_ 7.5) [33]

### Comments

A number of tyrosine kinase inhibitors targeting RET have been described [43].

## Type XV RTKs: RYK

### Overview

The ‘related to tyrosine kinase receptor’ (Ryk) is structurally atypical of the family of RTKs, particularly in the activation and ATP‐binding domains. RYK has been suggested to lack kinase activity and appears to be involved, with FZD8, in the Wnt signalling system [152].

**Table:** 

Nomenclature	receptor‐like tyrosine kinase
HGNC, UniProt	RYK, P34925
EC number	2.7.10.1
Common abreviation	RYK

### Comments

Thus far, no selective RYK inhibitors have been described.

## Type XVI RTKs: DDR (collagen receptor) family

### Overview

Discoidin domain receptors 1 and 2 (DDR1 and DDR2) are structurally‐related membrane protein tyrosine kinases activated by collagen. Collagen is probably the most abundant protein in man, with at least 29 families of genes encoding proteins, which undergo splice variation and post‐translational processing, and may exist in monomeric or polymeric forms, producing a triple‐stranded, twine‐like structure. In man, principal family members include COL1A1(COL1A1, P02452), COL2A1(COL2A1, P02458), COL3A1(COL3A1, P02461) and COL4A1(COL4A1, P02462).

**Table:** 

Nomenclature	discoidin domain receptor tyrosine kinase 1	discoidin domain receptor tyrosine kinase 2
HGNC, UniProt	DDR1, Q08345	DDR2, Q16832
EC number	2.7.10.1	2.7.10.1
Common abreviation	DDR1	DDR2

### Comments

The tyrosine kinase inhibitors of DDR, imatinib and nilotinib, were identified from proteomic analysis [34]. Other collagen receptors include glycoprotein VI (Q9HCN6), leukocyte‐associated immunoglobulin‐like receptor 1 (Q6GTX8), leukocyte‐associated immunoglobulin‐like receptor 2 (Q6ISS4) and osteoclast‐associated immunoglobulin‐like receptor (Q8IYS5).

## Type XVII RTKs: ROS receptors

**Table:** 

Nomenclature	c‐ros oncogene 1, receptor tyrosine kinase
HGNC, UniProt	ROS1, P08922
EC number	2.7.10.1
Common abreviation	ROS

### Comments

crizotinib is a tyrosine kinase inhibitor, anti‐cancer drug targeting ALK and ROS1.

## Type XVIII RTKs: LMR family

### Overview

The LMR kinases are unusual amongst the RTKs in possessing a short extracellular domain and extended intracellular domain (hence the ‘Lemur’ name reflecting the long tail). A precise function for these receptors has yet to be defined, although LMR1 was identified as a potential marker of apoptosis [48], giving rise to the name AATYK (Apoptosis‐associated tyrosine kinase); while over‐expression induces differentiation in neuroblastoma cells [155].

**Table:** 

Nomenclature	apoptosis associated tyrosine kinase	lemur tyrosine kinase 2	lemur tyrosine kinase 3
HGNC, UniProt	AATK, Q6ZMQ8	LMTK2, Q8IWU2	LMTK3, Q96Q04
EC number	2.7.11.1	2.7.11.1	2.7.11.1
Common abreviation	Lmr1	Lmr2	Lmr3

### Comments

As yet no selective inhibitors of the LMR family have been described.

## Type XIX RTKs: Leukocyte tyrosine kinase (LTK) receptor family

### Overview

The LTK family appear to lack endogenous ligands. LTK is subject to tissue‐specific splice variation, which appears to generate products in distinct subcellular locations. ALK fusions created by gene translocations and rearrangements are associated with many types of cancer, including large cell lymphomas, inflammatory myofibrilastic tumours and non‐small cell lung cancer [138].

**Table:** 

Nomenclature	leukocyte receptor tyrosine kinase	ALK receptor tyrosine kinase
HGNC, UniProt	LTK, P29376	ALK, Q9UM73
EC number	2.7.10.1	2.7.10.1
Common abreviation	LTK	ALK
Inhibitors	–	GSK‐1838705A (pIC_50_ 9.3) [159], compound 8e (pIC_50_ 9.1) [74], crizotinib (pIC_50_ 9) [30], NVP‐TAE684 (p*K*_d_ 9) [33], compound 25b (pIC_50_ 8.7) [53]
Selective inhibitors	–	ceritinib (pIC_50_ 9.7) [120]
Comments	–	crizotinib appears to be a selective ALK inhibitor acting on the tyrosine kinase activity [52]

## Type XX RTKs: STYK1

### Overview

Similar to the LMR RTK family, STYK1 has a truncated extracellular domain, but also displays a relatively short intracellular tail beyond the split kinase domain. STYK1 (also known as Novel Oncogene with Kinase‐domain, NOK) has been suggested to co‐localize with activated EGF receptor [38].

**Table:** 

Nomenclature	serine/threonine/tyrosine kinase 1
HGNC, UniProt	STYK1, Q6J9G0
EC number	2.7.10.2
Common abreviation	STYK1

### Comments

As yet, no selective inhibitors of STYK1 have been described.

### Further reading on Receptor tyrosine kinases (RTKs)

Alvarez‐Aznar A et al. (2017) VEGF Receptor Tyrosine Kinases: Key Regulators of Vascular Function. Curr Top Dev Biol 123: 433‐482 [PMID:28236974]

Bergeron JJ et al. (2016) Spatial and Temporal Regulation of Receptor Tyrosine Kinase Activation and Intracellular Signal Transduction. Annu Rev Biochem 85: 573‐97 [PMID:27023845]

Carvalho S et al. (2016) Immunotherapy of cancer: from monoclonal to oligoclonal cocktails of anti‐cancer antibodies: IUPHAR Review 18. Br J Pharmacol 173: 1407‐24 [PMID:26833433]

De Silva DM et al. (2017) Targeting the hepatocyte growth factor/Met pathway in cancer. Biochem Soc Trans [PMID:28673936]

Eklund, L et al. (2017) Angiopoietin‐Tie signalling in the cardiovascular and lymphatic systems. Clin Sci (Lond) 131 87‐103 [PMID:27941161]

Katayama, R. (2017) Therapeutic strategies and mechanisms of drug resistance in anaplastic lymphoma kinase (ALK)‐rearranged lung cancer. Pharmacol Ther [PMID:28185914]

Kazlauskas, A. (2017) PDGFs and their receptors. Gene 614 1‐7 [PMID:28267575]

Ke, EE et al. (2016) EGFR as a Pharmacological Target in EGFR‐Mutant Non‐Small‐Cell Lung Cancer: Where Do We Stand Now?. Trends Pharmacol Sci 37: 887‐903 [PMID:27717507]

Kuwano, M et al. (2016) Overcoming drug resistance to receptor tyrosine kinase inhibitors: Learning from lung cancer. Pharmacol Ther 161: 97‐110 [PMID:27000770]

Lee, DH. (2017) Treatments for EGFR‐mutant non‐small cell lung cancer (NSCLC): The road to a success, paved with failures. Pharmacol Ther 174: 1‐21 [PMID:28167215]

Nelson, KN et al. (2017) Receptor Tyrosine Kinases: Translocation Partners in Hematopoietic Disorders. Trends Mol Med 23: 59‐79 [PMID:27988109]

Simons M et al. (2016) Mechanisms and regulation of endothelial VEGF receptor signalling. Nat Rev Mol Cell Biol 17: 611‐25 [PMID:27461391]

Stricker S et al. (2017) ROR‐Family Receptor Tyrosine Kinases. Curr Top Dev Biol 123: 105‐142 [PMID:28236965]

Tan, AC et al. (2017) Exploiting receptor tyrosine kinase co‐activation for cancer therapy. Drug Discov Today 22: 72‐84 [PMID:27452454]

## Receptor serine/threonine kinase (RSTK) family

### Overview

Receptor serine/threonine kinases (RTSK), EC 2.7.11.30, respond to particular cytokines, the transforming growth factor *β*(TGF*β*) and bone morphogenetic protein (BMP) families, and may be divided into two subfamilies on the basis of structural similarities. Agonist binding initiates formation of a cell‐surface complex of type I and type II RSTK, possibly heterotetrameric, where where both subunits express serine/threonine kinase activity. The type I receptor serine/threonine kinases are also known as activin receptors or activin receptor‐like kinases, ALKs, for which a systematic nomenclature has been proposed (ALK1‐7). The type II protein phosphorylates the kinase domain of the type I partner (sometimes referred to as the signal propagating subunit), causing displacement of the protein partners, such as the FKBP12 FK506‐binding protein FKBP1A(P62942) and allowing the binding and phosphorylation of particular members of the Smad family. These migrate to the nucleus and act as complexes to regulate gene transcription. Type III receptors, sometimes called co‐receptors or accessory proteins, regulate the signalling of the receptor complex, in either enhancing (for example, presenting the ligand to the receptor) or inhibitory manners. TGF*β* family ligand signalling may be inhibited by endogenous proteins, such as follistatin(FST, P19883), which binds and neutralizes activins to prevent activation of the target receptors.

Endogenous agonists, approximately 30 in man, are often described as paracrine messengers acting close to the source of production. They are characterized by six conserved cysteine residues and are divided into two subfamilies on the basis of sequence comparison and signalling pathways activated, the TGF*β*/activin/nodal subfamily and the BMP/GDF (growth/differentiation factor)/MIS (Müllerian inhibiting substance) subfamily. Ligands active at RSTKs appear to be generated as large precursors which undergo complex maturation processes [103]. Some are known to form disulphide‐linked homo‐ and/or heterodimeric complexes. Thus, inhibins are *α* subunits linked to a variety of *β* chains, while activins are combinations of *β* subunits.

## Type I receptor serine/threonine kinases

### Overview

The type I receptor serine/threonine kinases are also known as activin receptors or activin receptor‐like kinases, ALKs, for which a systematic nomenclature has been proposed (ALK1‐7).

**Table:** 

Nomenclature	activin A receptor type IL	activin A receptor type 1	bone morphogenetic protein receptor type IA	activin A receptor type 1B	transforming growth factor beta receptor 1	bone morphogenetic protein receptor type IB	activin A receptor type 1C
HGNC, UniProt	ACVRL1, P37023	ACVR1, Q04771	BMPR1A, P36894	ACVR1B, P36896	TGFBR1, P36897	BMPR1B, O00238	ACVR1C, Q8NER5
Common abreviation	ALK1	ALK2	BMPR1A	ALK4	TGFBR1	BMPR1B	ALK7
Inhibitors		ML347 (pIC_50_ 7.5) [41]		–	LY2109761 (p*K*_i_ 7.4) [126], compound 15b (pIC_50_ 7.1) [102]		–
Selective inhibitors	–	–	–	EW‐7197 (pIC_50_ 7.9) [82]	EW‐7197 (pIC_50_ 8) [82]	–	–

## Type II receptor serine/threonine kinases

**Table:** 

Nomenclature	activin A receptor type 2A	activin A receptor type 2B	anti‐Mullerian hormone receptor type 2	bone morphogenetic protein receptor type 2	transforming growth factor beta receptor 2
HGNC, UniProt	ACVR2A, P27037	ACVR2B, Q13705	AMHR2, Q16671	BMPR2, Q13873	TGFBR2, P37173
Common abreviation	ActR2	ActR2B	MISR2	BMPR2	TGFBR2
Antibodies	–	bimagrumab (p*K*_d_ 11.8) [13]	–	–	–

## Type III receptor serine/threonine kinases

**Table:** 

Nomenclature	transforming growth factor beta receptor 3
HGNC, UniProt	TGFBR3, Q03167
Common abreviation	TGFBR3

## RSTK functional heteromers

### Overview

For the receptors listed on this page, the exact combination of subunits forming the functional heteromeric receptors is unknown.

**Table:** 

Nomenclature	Transforming growth factor *β* receptor	Bone morphogenetic protein receptors
Subunits	transforming growth factor beta receptor 1 (Type I), transforming growth factor beta receptor 3 (Type III), transforming growth factor beta receptor 2 (Type II)	bone morphogenetic protein receptor type IB (Type I), activin A receptor type 2B (Type II), activin A receptor type 2A (Type II), activin A receptor type IL (Type I), activin A receptor type 1 (Type I), bone morphogenetic protein receptor type IA (Type I), bone morphogenetic protein receptor type 2 (Type II)
Coupling	Smad2, Smad3 [135, 167]	Smad1, Smad5, Smad8 [153, 167]
Endogenous agonists	TGF*β*1 (TGFB1, P01137), TGF*β*2 (TGFB2, P61812), TGF*β*3 (TGFB3, P10600)	BMP‐10 (BMP10, O95393), BMP‐2 (BMP2, P12643), BMP‐4 (BMP4, P12644), BMP‐5 (BMP5, P22003), BMP‐6 (BMP6, P22004), BMP‐7 (BMP7, P18075), BMP‐8A (BMP8A, Q7Z5Y6), BMP‐8B (BMP8B, P34820), BMP‐9 (GDF2, Q9UK05)

**Table:** 

Nomenclature	Growth/differentiation factor receptors	Activin receptors	Anti‐Müllerian hormone receptors
Subunits	transforming growth factor beta receptor 1 (Type I), bone morphogenetic protein receptor type IB (Type I), activin A receptor type 2B (Type II), activin A receptor type 2A (Type II), activin A receptor type 1C (Type I), bone morphogenetic protein receptor type IA (Type I), activin A receptor type 1B (Type I), bone morphogenetic protein receptor type 2 (Type II)	activin A receptor type 2B (Type II), activin A receptor type 2A (Type II), activin A receptor type 1C (Type I), activin A receptor type 1B (Type I)	anti‐Mullerian hormone receptor type 2 (Type II), bone morphogenetic protein receptor type IB (Type I), activin A receptor type 1 (Type I), bone morphogenetic protein receptor type IA (Type I)
Coupling	Smad1, Smad5, Smad8 [135, 167]	Smad2, Smad3 [167]	Smad1, Smad5, Smad8 [135, 167]
Endogenous agonists	growth/differentiation factor‐1 (GDF1, P27539), growth/differentiation factor‐10 (GDF10, P55107), growth/differentiation factor‐3 (GDF3, Q9NR23), growth/differentiation factor‐7 (GDF7, Q7Z4P5), growth/differentiation factor‐9 (GDF9, O60383)	activin A (INHBA, P08476), activin AB (INHBA INHBB, P08476 P09529), activin B (INHBB, P09529), inhibin A (INHA INHBA, P05111 P08476)	Müllerian inhibiting substance (AMH, P03971)
Comments	–	Activin receptors are heteromeric complexes comprising activin receptor type I and type II subunits.	–

Comments on Receptor serine/threonine kinase (RSTK) family: A number of endogenous inhibitory ligands have been identified for RSTKs, including BMP‐3(BMP3, P12645), inhibin *α*(INHA, P05111), inhibin *β*C(INHBC, P55103) and inhibin *β*E(INHBE, P58166).

An appraisal of small molecule inhibitors of TGF*β* and BMP signalling concluded that TGF*β* pathway inhibitors were more selective than BMP signalling inhibitors [187]. The authors confirmed the selectivity of TGF‐beta RI inhibitor III to inhibit TGF*β* signalling through ALK4, ALK5, ALK7 [31]. Dorsomorphin inhibits BMP signalling through ALK2 and ALK3, it also inhibits AMP kinase [207].

Smads were identified as mammalian orthologues of Drosophila genes termed "mothers against decapentaplegic" and may be divided into Receptor‐regulated Smads (R‐Smads, including Smad1, Smad2, Smad3, Smad5 and Smad8), Co‐mediated Smad (Co‐Smad, Smad4) and Inhibitory Smads (I‐Smad, Smad6 and Smad7). R‐Smads form heteromeric complexes with Co‐Smad. I‐Smads compete for binding of R‐Smad with both receptors and Co‐Smad.

**Table:** 

Nomenclature	HGNC gene symbol	Uniprot ID	Other names
Smad1	SMAD1	Q15797	JV4‐1, MADH1, MADR1
Smad2	SMAD2	Q15796	JV18‐1, MADH2, MADR2
Smad3	SMAD3	P84022	HsT17436, JV15‐2, MADH3
Smad4	SMAD4	Q13485	DPC4, MADH4
Smad5	SMAD5	Q99717	Dwfc, JV5‐1, MADH5
Smad6	SMAD6	O43541	HsT17432, MADH6, MADH7
Smad7	SMAD7	O15105	MADH7, MADH8
Smad8	SMAD9	O15198	MADH6, MADH9

### Further reading on Receptor serine/threonine kinase (RSTK) family

Budi EH et al. (2017) Transforming Growth Factor‐beta Receptors and Smads: Regulatory Complexity and Functional Versatility. Trends Cell Biol [PMID:28552280]

Chen W et al. (2016) Immunoregulation by members of the TGFbeta superfamily. Nat Rev Immunol 16: 723‐740 [PMID:27885276]

Heger J. (2016) Molecular switches under TGFbeta signalling during progression from cardiac hypertrophy to heart failure. Br J Pharmacol 173: 3‐14 [PMID:26431212]

Luo JY et al. (2015) Regulators and effectors of bone morphogenetic protein signalling in the cardiovascular system. J Physiol 593: 2995‐3011 [PMID:25952563]

Macias MJ et al. (2015) Structural determinants of Smad function in TGF‐beta signaling. Trends Biochem Sci 40: 296‐308 [PMID:25935112]

Morrell NW et al. (2016) Targeting BMP signalling in cardiovascular disease and anaemia. Nat Rev Cardiol 13: 106‐20 [PMID:26461965]

Neuzillet C et al. (2015) Targeting the TGFbeta pathway for cancer therapy. Pharmacol Ther 147: 22‐31 [PMID:25444759]

van der Kraan PM. (2017) The changing role of TGFbeta in healthy, ageing and osteoarthritic joints. Nat Rev Rheumatol 13: 155‐163 [PMID:28148919]

## Receptor tyrosine phosphatase (RTP) family

### Overview

Receptor tyrosine phosphatases (RTP) are cell‐surface proteins with a single TM region and intracellular phosphotyrosine phosphatase activity. Many family members exhibit constitutive activity in heterologous expression, dephosphorylating intracellular targets such as Src tyrosine kinase (SRC) to activate signalling cascades. Family members bind components of the extracellular matrix or cell‐surface proteins indicating a role in intercellular communication.

**Table:** 

Nomenclature	RTP Type A	RTP Type B	RTP Type C	RTP Type D	RTP Type E	RTP Type F	RTP Type G
HGNC, UniProt	PTPRA, P18433	PTPRB, P23467	PTPRC, P08575	PTPRD, P23468	PTPRE, P23469	PTPRF, P10586	PTPRG, P23470
Putative endogenous ligands	–	–	galectin‐1 (LGALS1, P09382) [188]	netrin‐G3 ligand (LRRC4B, Q9NT99) [94]	–	netrin‐G3 ligand (LRRC4B, Q9NT99) [94]	contactin‐3 (CNTN3, Q9P232), contactin‐4 (CNTN4, Q8IWV2), contactin‐5 (CNTN5, O94779), contactin‐6 (CNTN6, Q9UQ52) [13]
Inhibitors	–	–	–	–	–	illudalic acid (pIC_50_ 5.9) [107]	compound 1 (p*K*_i_ 5.6) [166]

**Table:** 

Nomenclature	RTP Type H	RTP Type J	RTP Type K	RTP Type M	RTP Type N	RTP Type N2	RTP Type O
HGNC, UniProt	PTPRH, Q9HD43	PTPRJ, Q12913	PTPRK, Q15262	PTPRM, P28827	PTPRN, Q16849	PTPRN2, Q92932	PTPRO, Q16827
Putative endogenous ligands	–	–	galectin‐3 (LGALS3, P17931), galectin‐3 binding protein (LGALS3BP, Q08380) [88]	–	–	–	–

**Table:** 

Nomenclature	RTP Type Q	RTP Type R	RTP Type S	RTP Type T	RTP Type U	RTP Type Z1
HGNC, UniProt	PTPRQ, Q9UMZ3	PTPRR, Q15256	PTPRS, Q13332	PTPRT, O14522	PTPRU, Q92729	PTPRZ1, P23471
Putative endogenous ligands	–	–	chondroitin sulphate proteoglycan 3 (NCAN, O14594), netrin‐G3 ligand (LRRC4B, Q9NT99) [94, 165]	–	–	contactin‐1 (CNTN1, Q12860), pleiotrophin (PTN, C9JR52) (acts as a negative regulator) [13, 128]

### Further reading on Receptor tyrosine phosphatase (RTP) family

He R et al. (2013) Small molecule tools for functional interrogation of protein tyrosine phosphatases. FEBS J. 280: 731‐50 [PMID:22816879]

Papadimitriou E et al. (2016) Pleiotrophin and its receptor protein tyrosine phosphatase beta/zeta as regulators of angiogenesis and cancer. Biochim Biophys Acta 1866: 252‐265 [PMID:27693125]

Stanford SM et al. (2017) Targeting Tyrosine Phosphatases: Time to End the Stigma. Trends Pharmacol Sci 38: 524‐540 [PMID:28412041]

## Tumour necrosis factor (TNF) receptor family

### Overview

The TNF receptor superfamily (TNFRSF, provisional nomenclature) displays limited homology beyond an extracellular domain rich in cysteine residues and is activated by at least 18 different human homologues of TNF referred to as the TNF superfamily (TNFSF). Some homologues lacking transmembrane and cytoplasmic domains function as decoy receptors binding ligand without inducing cell signalling. Many of these receptors and ligands function as multimeric entities. Signalling through these receptors is complex and involves interaction with cytoplasmic adaptor proteins (such as TRADD and TRAF1). Several of these receptors contain cytoplasmic motifs known as ‘death domains’, which upon activation serve to recruit death domain‐ and death effector domain‐containing proteins crucial for the initiation of an apoptotic response. Additional signalling pathways include the regulation of the nuclear factor *κ*B or mitogen‐activated protein kinase pathways. Pharmacological manipulation of these receptors is mainly enacted through chelating the endogenous agonists with humanised monoclonal antibodies (e.g. Infliximab or adalimumab) or recombinant fusion proteins of IgG and soluble receptors (e.g. etanercept). Some mutated forms of TNF ligands are capable of selecting for different receptor subtypes.

**Table:** 

Nomenclature	tumor necrosis factor receptor 1	tumor necrosis factor receptor 2	lymphotoxin *β* receptor	OX40	CD40	Fas	decoy receptor 3
Systematic nomenclature	TNFRSF1A	TNFRSF1B	TNFRSF3	TNFRSF4	TNFRSF5	TNFRSF6	TNFRSF6B
HGNC, UniProt	TNFRSF1A, P19438	TNFRSF1B, P20333	LTBR, P36941	TNFRSF4, P43489	CD40, P25942	FAS, P25445	TNFRSF6B, O95407
Adaptor proteins	TRADD	TRAF1, TRAF2, TRAF5	TRAF3, TRAF4, TRAF5	TRAF1, TRAF2, TRAF3, TRAF5	TRAF1, TRAF2, TRAF3, TRAF5, TRAF6	FADD	–
Common abreviation	TNFR1	TNFR2	–	–	–	–	–
Endogenous ligands	lymphotoxin‐*α* (LTA, P01374), tumour necrosis factor membrane form (TNF, P01375), tumour necrosis factor shed form (TNF, P01375)	lymphotoxin‐*α* (LTA, P01374), tumour necrosis factor membrane form (TNF, P01375)	LIGHT (TNFSF14, O43557), lymphotoxin *β*_2_*α*_1_ heterotrimer (LTA LTB, P01374 Q06643)	OX‐40 ligand (TNFSF4, P23510)	CD40 ligand (CD40LG, P29965)	Fas ligand (FASLG, P48023)	–
Inhibitors	–	–	–	compound 1 (pIC_50_ 5.9) [169]	–	–	–
Comments	–	–	–	The OX40/OX40L pair is involved in late T‐cell costimulatory signaling and both are transiently expressed following antigen recognition, and blocking OX40/OX40L is reported to prevent the development of disease in in vivo autoimmune and inflammatory disease models [191]	–	–	Decoy receptor for LIGHT (TNFSF14, O43557), TL1A (TNFSF15, O95150) and Fas ligand (FASLG, P48023).

**Table:** 

Nomenclature	CD27	CD30	4‐1BB	death receptor 4	death receptor 5	decoy receptor 1	decoy receptor 2
Systematic nomenclature	TNFRSF7	TNFRSF8	TNFRSF9	TNFRSF10A	TNFRSF10B	TNFRSF10C	TNFRSF10D
HGNC, UniProt	CD27, P26842	TNFRSF8, P28908	TNFRSF9, Q07011	TNFRSF10A, O00220	TNFRSF10B, O14763	TNFRSF10C, O14798	TNFRSF10D, Q9UBN6
Adaptor proteins	TRAF2, SIVA	TRAF1, TRAF2, TRAF3, TRAF5	TRAF1, TRAF2, TRAF3	FADD	FADD	–	–
Common abreviation	–	–	–	DR4	DR5	–	–
Endogenous ligands	CD70 (CD70, P32970)	CD30 ligand (TNFSF8, P32971)	4‐1BB ligand (TNFSF9, P41273)	TRAIL (TNFSF10, P50591)	–	–	–
Endogenous agonists	–	–	–	–	TRAIL (TNFSF10, P50591) [208]	–	–
Agonists	–	–	–	SC‐67655 [77]	–	–	–
Antibodies	–	brentuximab vedotin (Inhibition)	–	–	tigatuzumab (Agonist) (p*K*_d_∼8.5) [208]	–	–
Comments	–	–	–	–	–	Decoy receptor for TRAIL (TNFSF10, P50591).	Decoy receptor for TRAIL (TNFSF10, P50591).

**Table:** 

Nomenclature	receptor activator of NF‐kappa B	osteoprotegerin	death receptor 3	TWEAK receptor	TACI	BAFF receptor	herpes virus entry mediator
Systematic nomenclature	TNFRSF11A	TNFRSF11B	TNFRSF25	TNFRSF12A	TNFRSF13B	TNFRSF13C	TNFRSF14
HGNC, UniProt	TNFRSF11A, Q9Y6Q6	TNFRSF11B, O00300	TNFRSF25, Q93038	TNFRSF12A, Q9NP84	TNFRSF13B, O14836	TNFRSF13C, Q96RJ3	TNFRSF14, Q92956
Adaptor proteins	TRAF1, TRAF2, TRAF3, TRAF5, TRAF6	–	TRADD	TRAF1, TRAF2, TRAF3	TRAF2, TRAF5, TRAF6	TRAF3	TRAF2, TRAF3, TRAF5
Common abreviation	RANK	OPG	DR3	–	–	BAFF‐R	HVEM
Endogenous ligands	RANK ligand (TNFSF11, O14788)	–	TL1A (TNFSF15, O95150)	TWEAK (TNFSF12, O43508)	APRIL (TNFSF13, O75888), BAFF (TNFSF13B, Q9Y275)	BAFF (TNFSF13B, Q9Y275)	B and T lymphocyte attenuator (BTLA, Q7Z6A9), LIGHT (TNFSF14, O43557), lymphotoxin‐*α* (LTA, P01374)
Comments	–	Acts as a decoy receptor for RANK ligand (TNFSF11, O14788) and possibly for TRAIL (TNFSF10, P50591).	The only known TNFSF ligand for DR3 is TNF‐like protein 1A (TL1A) [218].	–	–	–	–

**Table:** 

Nomenclature	nerve growth factor receptor	B cell maturation antigen	glucocorticoid‐induced TNF receptor	toxicity and JNK inducer	RELT	death receptor 6
Systematic nomenclature	TNFRSF16	TNFRSF17	TNFRSF18	TNFRSF19	TNFRSF19L	TNFRSF21
HGNC, UniProt	NGFR, P08138	TNFRSF17, Q02223	TNFRSF18, Q9Y5U5	TNFRSF19, Q9NS68	RELT, Q969Z4	TNFRSF21, O75509
Adaptor proteins	TRAF2, TRAF4, TRAF6	TRAF1, TRAF2, TRAF3, TRAF5, TRAF6	TRAF1, TRAF2, TRAF3, SIVA	TRAF1, TRAF2, TRAF3, TRAF5	TRAF1	TRADD
Common abreviation	–	BCMA	GITR	TAJ	–	DR6
Endogenous ligands	NGF (NGF, P01138) (pIC_50_ 6) [97], BDNF (BDNF, P23560), neurotrophin‐3 (NTF3, P20783), neurotrophin‐4 (NTF4, P34130)	APRIL (TNFSF13, O75888), BAFF (TNFSF13B, Q9Y275)	TL6 (TNFSF18, Q9UNG2)	lymphotoxin‐*α* (LTA, P01374)	–	–
Comments	One of the two receptor types for the neurotrophins (factors that stimulate neuronal cell survival and differentiation). The other family of neurotrophin receptors are the Trk family of receptor tyrosine kinases.	–	–	Believed to be essential during embryonic development.	Abundant in hematologic tissues. Selective receptor for TNF receptor‐associated factor 1 (TRAF1). Activates the NF‐*κ*B pathway.	–

**Table:** 

Nomenclature	TNFRSF22	TNFRSF23	ectodysplasin A2 isoform receptor	ectodysplasin 1, anhidrotic receptor
Systematic nomenclature	–	–	TNFRS27	–
HGNC, UniProt	–	–	EDA2R, Q9HAV5	EDAR, Q9UNE0
Adaptor proteins	–	–	TRAF1, TRAF3, TRAF6	TRAF1, TRAF2, TRAF3
Endogenous ligands	–	–	ectodysplasin A2 (EDA, Q92838) [201]	ectodysplasin A1 (EDA, Q92838) [201]
Comments	Only identified in mouse to date. A potential decoy receptor for the cytotoxic ligand TNFSF10/TRAIL. Does not contain a cytoplasmic death domain so does not induce apoptosis, and does not activate the NF‐*κ*B signalling pathway.	Only identified in mouse to date. A potential decoy receptor for the cytotoxic ligand TNFSF10/TRAIL. Does not contain a cytoplasmic death domain so does not induce apoptosis, and does not activate the NF‐*κ*B signalling pathway.	Receptor for the EDA‐A2 isoform of ectodysplasin encoded by the anhidrotic ectodermal dysplasia (EDA) gene.	Cell surface receptor for ectodysplasin A (a morphogen involved in the development of ectodermal tissues, including skin, hair, nails, teeth, and sweat glands).

### Comments

TNFRSF1A is preferentially activated by the shed form of TNF ligand, whereas the membrane‐bound form of TNF serves to activate TNFRSF1A and TNFRSF1B equally. The neurotrophins nerve growth factor (NGF(NGF, P01138)), brain‐derived neurotrophic factor (BDNF(BDNF, P23560)), neurotrophin‐3(NTF3, P20783) (NTF3) and neurotrophin‐4(NTF4, P34130) (NTF4) are structurally unrelated to the TNF ligand superfamily but exert some of their actions through the “low affinity nerve growth factor receptor” (NGFR (TNFRSF16)) as well as through the TRK family of receptor tyrosine kinases. The endogenous ligands for EDAR and EDA2R are, respectively, the membrane (Q92838[1‐391]) and secreted (Q92838[160‐391]) isoforms of Ectodysplasin‐A (EDA, Q92838).

### Further reading on Tumour necrosis factor (TNF) receptor family

Blaser H et al. (2016) TNF and ROS Crosstalk in Inflammation. Trends Cell Biol 26: 249‐61 [PMID:26791157]

Croft M et al. (2017) Beyond TNF: TNF superfamily cytokines as targets for the treatment of rheumatic diseases. Nat Rev Rheumatol 13: 217‐233 [PMID:28275260]

Kalliolias GD et al. (2016) TNF biology, pathogenic mechanisms and emerging therapeutic strategies. Nat Rev Rheumatol 12: 49‐62 [PMID:26656660]

Olesen CM et al. (2016) Mechanisms behind efficacy of tumor necrosis factor inhibitors in inflammatory bowel diseases. Pharmacol Ther 159: 110‐9 [PMID:26808166]

von Karstedt S et al. (2017) Exploring the TRAILs less travelled: TRAIL in cancer biology and therapy. Nat Rev Cancer 17: 352‐366 [PMID:28536452]
